# Acupuncture for behavioral changes of experimental depressive disorder: a systematic review and meta-analysis

**DOI:** 10.1038/s41598-017-09712-1

**Published:** 2017-08-29

**Authors:** Ren-zhong Kou, Hong Chen, Mei-ling Yu, Tian-cheng Xu, Shu-ping Fu, Sheng-feng Lu

**Affiliations:** 1 The No.2 Clinical Medicine College, Nanjing University of Chinese Medicine, Nanjing, 210023 China; 2 Key Laboratory of Acupuncture and Medicine Research of Ministry of Education, Nanjing University of Chinese Medicine, Nanjing, 210023 China

## Abstract

Acupuncture is considered to be a promising alternative therapy for depression. Nevertheless, up to now, it remains controversial on the effects exerted by acupuncture on behavioral changes in depression models. Consequently, it’s necessary to develop a systematic review and meta-analysis to assess the effect of acupuncture for model rats of depression. Ultimately, 90 studies involving 1861 models were identified. Behavioral indicators including the number of crossings (NC) and the number of rearings (NR) in open field test (OFT), the capacity of sucrose intake (CSI) and the rate of sucrose intake (RSI) in sucrose intake test (SIT), final weight (FW) and gain weight (GW) were employed as main outcomes in depression model rats. The pooled results showed that acupuncture had not less effect than western medicine on improving NC, NR, FW, GW, RSI (P > 0.05). However, the CSI improvement was poorer compared with west medicine (P < 0.05). In conclusion, acupuncture has not less effect on behavior changes than western medicine, including improving NC, NR, RSI, FW and GW in depression models.

## Introduction

Major Depressive Disorder (MDD) is one of the most common and costly diseases. It is the third most common reason for primary care consultations and will be the second leading cause of disability by 2020 according to the current estimates by WHO^1^. Nevertheless, the available treatments, especially anti-depressants which are the first-line treatment in medical care, are limited by poor efficacy, lagged therapeutic time and undesirable side effects^2^. New strategies which are more effective and less adverse effects for treating depression are urgently needed.

Acupuncture is widely used in psychiatric conditions’ treatment in China for thousands of years^3^. Recent studies demonstrate that depressive patients can benefit from acupuncture therapy in both clinical effectiveness and cost-effectiveness^1, 4^. Although confirmed to be a generally safe and well tolerated therapy^5^, the mechanisms underlying the effects of acupuncture on depression have not been fully explained. Therefore, many studies, in which rodents are frequently utilized, have explored mechanisms of antidepressant effects of acupuncture. Given that there were not valid biomarkers accepted as effective links between clinical symptoms and animal end points^6^, depression-like behaviors which simulate the core symptoms in depression are frequently utilized to judge whether the models are successful and whether the interventions are effective^3, 7–10^. However, effects of acupuncture on behavioral tests including open field test (OFT), sucrose intake test (SIT), and body weight in depression models are controversial in different studies^3, 7, 8^. To some extent, these controversial impede the application of these end points.

Due to its potential values, such as promoting the conduct and report of basic researches and providing some guidance to translate the achievements of basic researches to clinical application in acupuncture for depression, and so on, we conducted this systematic review and meta-analysis to evaluate effects of acupuncture on behavioral changes in depression model rats.

## Results

### Study inclusion

**Figure 1:**
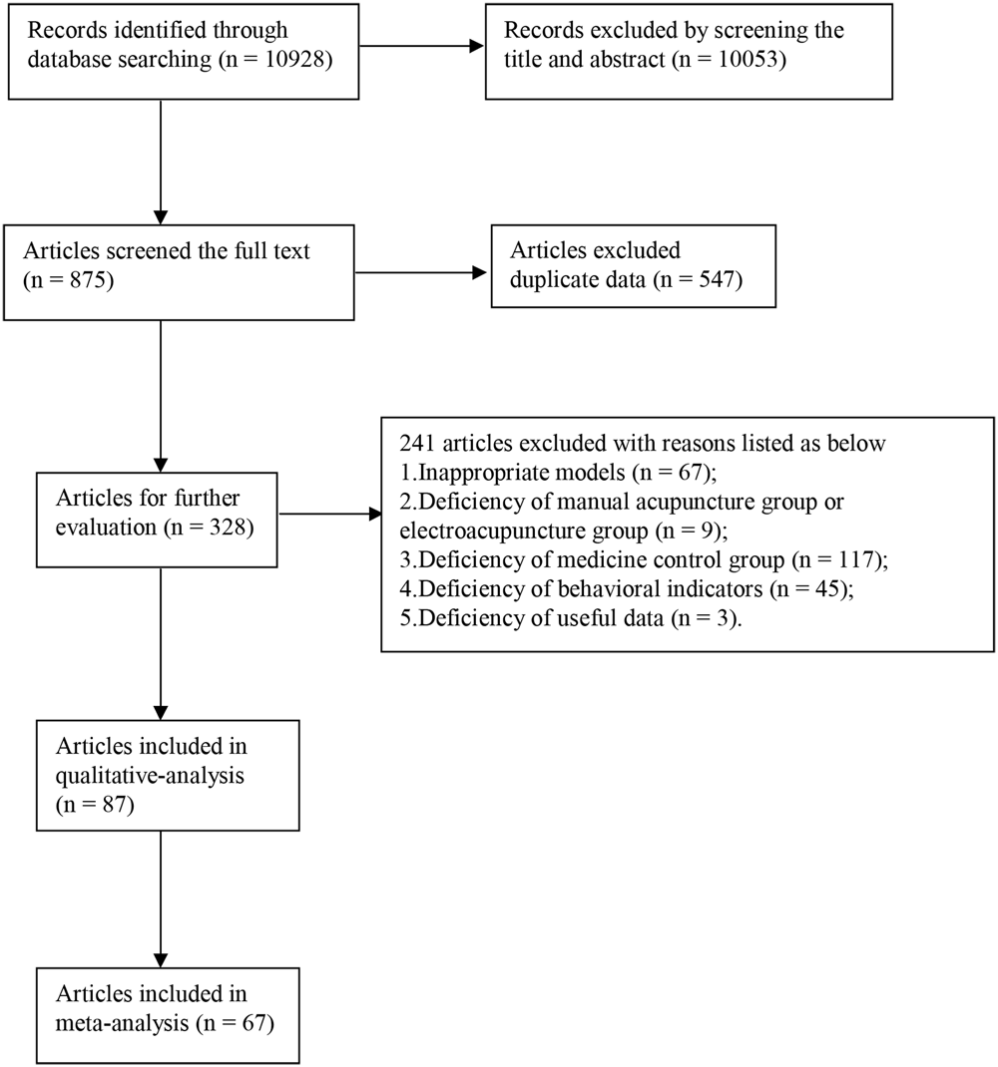
Flowchart of the articles selection process.

We screened 10928 potentially relevant articles from seven databases and 10053 articles were excluded by going through titles and abstracts with at least one of the following reasons: (1) the type of study is not animal research (such as case report, clinical trial, review, or meta-analysis), (2) the animal models are not depression models, or the animals in studies are not rats. Full texts of 875 remained articles were assessed for eligibility and 788 of them were excluded according to our inclusion and exclusion criteria. Finally, 87^11–97^articles met the inclusion criteria and were included in the systematic review, of which studies, 67^11, 13–15, 17, 18, 21–30, 32–38, 40, 42, 45–47, 51, 53–56, 59–74, 76–78, 80, 81, 83–85, 87–95, 97^ provided raw data were applied in meta-analysis. The process of literature search is displayed in the flowchart (as shown in Fig. 1).

### Study characteristics

The characteristics of each research are summarized in Table 1. We split one article^77^ into three studies and another one^39^ into two studies due to each study selected different depression models. Therefore, 90 studies in 87 articles were included in this review. A majority of studies selected chronic unpredictable mild stress (CUMS) or CUMS combined with solitary models. Besides chronic forced swim stress (CFSS) models^11^, intracerebroventricular injection of ibotenic acid (IA) models^22^, chronic restraint stress (CRS) models^26^, Learned Helpless (LH) models^39^, intracerebroventricular injection of L-α-aminoadipic acid (L-AAA) models^64^, intracerebroventricular injection of Excitatory Amino Acid Transporter 1 (EAAT1) antagonist models^77^, prefrontal cortex injection of Excitatory Amino Acid Transporter 2 (EAAT2) antagonist models^77^, pre-shocked animal (PSA) models^95^ were also used in some studies. Totally 1861 depression model rats were included, with the average number of approximately 21 per study, ranging from 14 to 40. The rats’ species included Sprague-Dawley (SD) and Wistar rats. The weight of rats varied between 100–300 g. Eighty-nine studies mentioned randomization and eleven of them selected random number table. None of the studies mentioned blinding. Fifty-eight studies chose intervention and modeling at the same time and the rest chose intervention after or in the process of modeling. Sixty-six studies selected electroacupuncture as intervention and the rest selected manual acupuncture. Only 4 studies did not mention stimulus parameter, the duration of one session varied in 10 min, 15 min, 20 min, 30 min, 40 min, 60 min, the number of sessions varied from 5 to 35 and treatment course varied between 5d-42d. Details of western medicine group and behavioral indicators are also summarized in Table 1. We also summarized selected acupoints and methods for selection of acupoints in Table S1 and we hope it can be valuable for planning researches in the future.

**Table 1:** Characteristics of the studies included.

Research	Model	Species	Sex	Weight (g)	N	Randomization	Blinding	Inter–vention Time	Inter–ventions	Acupoints	Stimulus parameter	Duration of one session	The number of sessions	Treatment course	Western medicine	Indicators
Bao 2014^11^	CFSS	SD rats	Male	180 ± 10	10/10	mention	not mention	SAME	EA	GV20,GV29	mention	10 min	14	14d	Fluoxetine	OFT:NC,NR; W:FW; SI:CSI.
Bao 2014#^12^	CUMS	SD rats	Male	200 ± 20	12/12	mention	not mention	SAME	EA	GV20,GV29	mention	20 min	21	21d	Fluoxetine	W:FW;
Chen 2011^13^	CUMS	SD rats	Female	180–220	10/10	Random number table	not mention	NOT SAME	EA	GV20,GV24	mention	30 min	14	14d	Fluoxetine	OFT:NC,NR.
Cheng 2015^14^	CUMS	SD rats	Male	200	8/8	mention	not mention	SAME	EA	GV20,GV29	mention	30 min	21	21d	Fluoxetine	W:FW; SI:CSI.
Dai 2010^15^	CUMS	SD rats	Male	200 ± 20	13/13	mention	not mention	SAME	EA	GV20,GV29	mention	20 min	21	21d	Fluoxetine	OFT:NC,NR.
Deng 2013^16^	CUMS & Solitary	SD rats	Male	160–180	12/12	mention	not mention	SAME	EA	GV20,GV29	mention	20 min	21	21d	Fluoxetine	OFT:NC,NR;W:GW; SI:CSI.
Ding 2016^17^	CUMS	SD rats	Male	200–210	8/8	mention	not mention	NOT SAME	MA	GV20,GV14	mention	20 min	18	21d	Fluoxetine	OFT:NC,NR;W:FW,GW; SI:RSI.
Duan 2008^18^	CUMS & Solitary	Wistar rats	Male	150–180	10/10	mention	not mention	SAME	EA	GV20,GV29,EX-HNl	mention	20 min	21	21d	Fluoxetine	OFT:NC,NR; W:FW.
Duan 2016^19^	CUMS	SD rats	Male	150 ± 10	16/16	mention	not mention	NOT SAME	EA	GV20,GV29	mention	30 min	28	28d	Fluoxetine	OFT:NC,NR;W:FW;
Duan 2016#^20^	CUMS & Solitary	Wistar rats	Male	180–200	10/10	mention	not mention	NOT SAME	EA	GV20,GV29	mention	20 min	28	28d	Fluoxetine	OFT:NC,NR;W:FW; SI:RSI.
Fan 2013^21^	CUMS & Solitary	SD rats	Female/Male	170–220	10/10	Random number table	not mention	SAME	MA	EX-HNl,PC6,SP6	mention	30 min	21	21d	Fluoxetine	OFT:NC,NR; |W:FW; SI:CSI.
Fan 2016^22^	IA	SD rats	Male	180–220	8/8	mention	not mention	NOT SAME	EA	LI4,LR3	mention	15 min	21	21d	Riluzole	OFT:NC,NR;
Fan 2016#^23^	CUMS	SD rats	Male	180–220	8/8	mention	not mention	NOT SAME	EA	LI4,LR3	mention	15 min	21	21d	Riluzole	OFT:NC,NR;W:FW; SI:CSI.
Fan 2016*^24^	CUMS	SD rats	Male	180–220	8/8	mention	not mention	NOT SAME	MA	LI4,LR3	not mention	not mention	21	21d	Riluzole	OFT:NC,NR;W:FW; SI:CSI.
Fu 2008^25^	CUMS	SD rats	Female/Male	180–200	8/8	mention	not mention	SAME	EA	LI4,LR3	mention	15 min	21	21d	Fluoxetine	OFT:NC,NR; W:FW,GW.
Guo 2016^26^	CRS	SD rats	Male	200 ± 20	8/8	mention	not mention	SAME	MA	GV20,GV29,SP6	mention	20 min	28	28d	Fluoxetine	OFT:NC,NR;W:FW; SI:CSI,RSI.
Hu 2013^27^	CUMS & Solitary	SD rats	Male	200 ± 20	8/8	mention	not mention	SAME	MA	GV20,PC6	mention	10 min	14	28d	Fluoxetine	OFT:NC,NR; W:FW; SI:RSI
Hu 2014^28^	CUMS & Solitary	SD rats	Male	200 ± 20	10/10	Random number table	not mention	SAME	EA	GV20,GV29	mention	30 min	28	28d	Fluoxetine	OFT:NC,NR; W:FW; SI:CSI
Huang 2005^29^	CUMS & Solitary	Wistar rats	Male	200–230	7/7	mention	not mention	NOT SAME	EA	PC6,CV17	mention	30 min	21	21d	Amitriptyline	OFT:NC,NR; W:GW; SI:CSI.
Ji 2013^30^	CUMS & Solitary	SD rats	Male	200 ± 20	12/12	mention	not mention	SAME	EA	GV20,GV29	mention	20 min	21	21d	Fluoxetine	OFT:NC,NR.
Jia 2005^31^	CUMS & Solitary	SD rats	Male	160–180	10/10	mention	not mention	SAME	EA	GV20,GV29	mention	20 min	21	21d	Fluoxetine	OFT:NC,NR;W:FW; SI:CSI.
Jiang 2007^32^	CUMS	SD rats	Female	180–220	8/8	mention	not mention	NOT SAME	EA	GV20,GV29	mention	10 min	21	21d	Fluoxetine	OFT:NC,NR; W:FW; SI:CSI.
Jiang 2013^33^	CUMS & Solitary	SD rats	Female	220–250	18/18	mention	not mention	NOT SAME	EA	LI4,LR3	mention	15–20 min	14	21d	Riluzole	OFT:NC,NR; W:FW;SI:RSI.
Jiao 2008^34^	CUMS & Solitary	SD rats	Male	180–200	12/12	mention	not mention	SAME	EA	GV20,GV29	mention	30 min	21	21d	Fluoxetine	OFT:NC,NR;W:FW.
Jin 2015^35^	CUMS & Solitary	SD rats	Male	200 ± 20	9/9	mention	not mention	SAME	MA	GV20,PC6	mention	10 min	14	28d	Fluoxetine	OFT:NC,NR;W:FW; SI:CSI.
Jing 2016^36^	CUMS & Solitary	SD rats	Male	200 ± 10	8/8	random number table	not mention	SAME	EA	GV20,GV29	mention	20 min	21	21d	Fluoxetine	OFT:NC,NR;
Li 2007^37^	CUMS	SD rats	Male	200 ± 20	8/8	mention	not mention	SAME	EA	GV20,GV29	mention	20 min	21	21d	Fluoxetine	OFT:NC,NR; W:FW,GW; SI:CSI.
Li 2008^38^	CUMS	SD rats	Male	200 ± 20	13/13	Random number table	not mention	SAME	EA	GV20,GV29	mention	20 min	21	21d	Fluoxetine	OFT:NC,NR; W:FW,GW.
Li 2011^39^	LH	Wistar rats/SD rats	Male	200 ± 20	20/15	mention	not mention	NOT SAME	EA	GV20,GB34	mention	60 min	5	5d	Chlorimipramine	OFT:NC,NR.
Li 2011#^39^	CUMS	Wistar rats/SD rats	Male	200 ± 20	10/10	mention	not mention	NOT SAME	EA	GV20,GB34	not mention	not mention	14	28d	Chlorimipramine	OFT:NC,NR.
Li 2011&^40^	CUMS	SD rats	Male	180–220	15/15	mention	not mention	NOT SAME	EA	GV20,GV29	mention	15 min	7	7d	Fluoxetine	OFT:NC,NR; W:FW; SI:CSI.
Li 2014^41^	CUMS & Solitary	SD rats	Male	200 ± 10	12/12	mention	not mention	SAME	EA	GV20,GV29	mention	20 min	21	21d	Fluoxetine	OFT:NC,NR.
Lin 2008^42^	CUMS & Solitary	SD rats	Male	200–230	10/10	mention	not mention	SAME	EA	GV20,SP6	mention	20 min	21	21d	Fluoxetine	OFT:NC,NR; W:GW.
Liu 2005^43^	CUMS	SD rats	Male	100–120	8/8	mention	not mention	NOT SAME	EA	GV20,GB34	mention	30 min	7	14d	Chlorimipramine	OFT:NC,NR;W:GW.
Liu 2008^44^	CUMS	SD rats	Male	200 ± 20	8/8	mention	not mention	NOT SAME	EA	GV20,GB34	mention	30 min	10	21d	Chlorimipramine	OFT:NC,NR.
Liu 2009^45^	CUMS & Solitary	SD rats	Male	around 250	10/10	mention	not mention	SAME	EA	GV20,GV29,ST36,SP6	mention	20 min	21	21d	Fluoxetine	OFT:NC,NR; W:FW,GW SI:RSI
Liu 2012^46^	CUMS	SD rats	Male	200–250	8/8	mention	not mention	NOT SAME	EA	LI4,LR3	mention	30 min	10	21d	Fluoxetine	OFT:NC,NR;x W:FW; SI:CSI.
Lu 2008^47^	CUMS & Solitary	SD rats	Male	200 ± 20	8/8	mention	not mention	SAME	EA	GV20,GV29,ST36,ST40	mention	15 min	11	22d	Maprotiline	OFT:NC,NR; W:FW; SI:CSI.
Lu 2013^48^	CUMS	SD rats	Male	180–200	10/10	mention	not mention	SAME	MA	GV20,PC6	mention	10 min	14	28d	Paroxetine	OFT:NC,NR;W:FW; SI:RSI.
Lu 2016^49^	CUMS	SD rats	Male	180–200	8/8	mention	not mention	SAME	MA	GV20,PC6	mention	10 min	14	28d	Fluoxetine	OFT:NC,NR;W:FW; SI:CSI.
Lu 2016#^50^	CUMS	SD rats	Male	180–200	8/8	mention	not mention	SAME	MA	GV20,PC6	mention	10 min	14	28d	Fluoxetine	OFT:NC,NR;W:FW; SI:RSI.
Luo 2016^51^	CUMS	SD rats	Male	260–300	10/10	mention	not mention	NOT SAME	EA	LI4,LR3	mention	30 min	35	35d	Riluzole	OFT:NC,NR;SI:RSI.
Ma 2016^52^	CUMS	SD rats	Male	200 ± 15	12/12	random number table	not mention	SAME	EA	GV20,GV29	mention	20 min	21	21d	Fluoxetine	OFT:NC,NR;
Mo 2014^53^	CUMS	SD rats	Male	200 ± 10	12/12	mention	not mention	SAME	EA	GV20,GV29	mention	20 min	21	21d	Fluoxetine	OFT:NC,NR.
Pan 2016^54^	CUMS	SD rats	Male/Female	180–230	10/10	mention	not mention	SAME	MA	GV20,PC6,SP6	mention	30 min	21	21d	Fluoxetine	OFT:NC,NR;W:FW; SI:CSI.
Qin 2010^55^	CUMS & Solitary	SD rats	Male	170 ± 10	10/10	mention	not mention	SAME	EA	GV20,GV29,ST25	mention	30 min	21	21d	Fluoxetine	OFT:NC,NR; W:FW.
Shao 2016^56^	CUMS & Solitary	SD rats	Male	200 ± 20	8/8	mention	not mention	SAME	MA	GV20,PC6	mention	10 min	14	28d	Fluoxetine	OFT:NC,NR;W:FW; SI:CSI.
Shi 2007^57^	CUMS	SD rats	Male	160–180	10/10	mention	not mention	SAME	EA	GV20,GV29	mention	20 min	21	21d	Fluoxetine	OFT:NC,NR;W:FW; SI:CSI.
Shi 2015^58^	CUMS & Solitary	SD rats	Male	200 ± 20	10/10	random number table	not mention	SAME	MA	GV20,GV29,GB20,BL23	mention	30 min	28	28d	Fluoxetine	OFT:NC,NR;
Song 2014^59^	CUMS & Solitary	SD rats	Male	200 ± 10	13/13	mention	not mention	SAME	EA	GV20,GV29	mention	20 min	21	21d	Fluoxetine	OFT:NC,NR; W:FW; SI:CSI
Song 2015^60^	CUMS & Solitary	SD rats	Male	180 ± 10	8/8	mention	not mention	SAME	MA	GV20,GV29	mention	20 min	21	21d	Fluoxetine	OFT:NC,NR;W:FW; SI:CSI.
Song 2016^61^	CUMS & Solitary	SD rats	Male	200 ± 20	8/8	mention	not mention	SAME	MA	GV20,GV14	mention	20 min	18	21d	Fluoxetine	OFT:NC,NR;W:FW; SI:RSI.
Song 2014#^62^	CUMS & Solitary	SD rats	Male	200 ± 10	8/8	mention	not mention	SAME	EA	GV20,GV29	mention	20 min	21	21d	Fluoxetine	OFT:NC,NR.
Sun 2003^63^	CUMS	SD rats	not mention	220–240	8/8	mention	not mention	NOT SAME	EA	GV20,ST36	mention	30 min	21	21d	Clomipramine	OFT:NC,NR.
Sun 2013^64^	L-AAA	SD rats	Female	220–250	9/9	mention	not mention	SAME	EA	LI4,LR3	mention	15 min	21	21d	Riluzole	OFT:NC,NR; W:FW; SI:RSI.
Sun 2014^65^	CUMS & Solitary	SD rats	Male	200 ± 20	10/10	mention	not mention	SAME	EA	GV20,GV29,PC6	mention	20 min	14	28d	Paroxetine	OFT:NC,NR.
Sun 2016^66^	CUMS	SD rats	Male	200 ± 20	8/8	mention	not mention	NOT SAME	EA	CV4, ST36	mention	30 min	14	14d	Fluoxetine	OFT:NC,NR;SI:RSI.
Tang 2013^67^	CUMS & Solitary	SD rats	Male	200 ± 20	10/10	mention	not mention	SAME	EA	GV20,GV29	mention	20 min	21	21d	Fluoxetine	OFT:NC,NR; W:FW; SI:CSI
Tang 2014^68^	CUMS & Solitary	SD rats	Male	180–220	10/10	Random number table	not mention	SAME	EA	GV20,GV29	mention	20 min	21	21d	Fluoxetine	OFT:NC,NR; W:FW; SI:CSI.
Teng 2013^69^	CUMS & Solitary	SD rats	Male	180 ± 20	12/12	mention	not mention	SAME	EA	GV20,GV29	mention	20 min	21	21d	Fluoxetine	OFT:NC,NR;W:FW; SI:CSI.
Wang 2008^70^	CUMS & Solitary	SD rats	not mention	160–190	10/10	Random number table	not mention	NOT SAME	EA	GV20,GV29,SP6	mention	15 min	10	21d	Clomipramine	OFT:NC,NR; W:FW,GW; SI:CSI.
Wang 2009^71^	CUMS	SD rats	Male	180–220	10/10	mention	not mention	NOT SAME	EA	GV20,GV29	mention	15 min	7	7d	Fluoxetine	OFT:NC,NR; W:FW; SI:CSI.
Wang 2010^72^	CUMS & Solitary	SD rats	Male	180–220	10/10	not mention	not mention	SAME	EA	GV20,LR3	mention	20 min	21	21d	Fluoxetine	OFT:NC,NR; W:FW; SI:CSI.
Wang 2013^73^	CUMS & Solitary	SD rats	Male	180–220	12/12	mention	not mention	SAME	EA	GV20,GV24	mention	15 min	7	7d	Fluoxetine	OFT:NC,NR; SI:CSI.
Wang 2014^74^	CUMS & Solitary	SD rats	Male	180 ± 20	12/12	mention	not mention	NOT SAME	MA	Ex-B2	not mention	not mention	28	14d	Fluoxetine	OFT:NC,NR; W:FW.
Wang 2016^75^	CUMS & Solitary	SD rats	Male	200 ± 20	12/12	random number table	not mention	SAME	EA	GV20,GV24	mention	20 min	21	21d	Fluoxetine	W:FW; SI:CSI.
Wu 2007^76^	CUMS	SD rats	Male	120–140	10/10	mention	not mention	NOT SAME	EA	ST36	mention	15 min	22	22d	Fluoxetine	OFT:NC,NR; W:GW.
Xiao 2014^77^	CUMS & Solitary	SD rats	Male	250–300	15/15	mention	not mention	NOT SAME	MA	LI4,LR3	mention	30 min	21	35d	Riluzole	W:FW; SI:RSI.
Xiao 2014#^77^	EAAT1	SD rats	Male	250–300	10/10	mention	not mention	NOT SAME	MA	LI4,LR3	mention	30 min	21	35d	Riluzole	OFT:NC; W:FW; SI:RSI.
Xiao 2014&^78^	CUMS & Solitary	SD rats	Female	220–250	20/20	mention	not mention	NOT SAME	EA	LI4,LR3	not mention	not mention	14	21d	Riluzole	SI:RSI
Xiao 2014*^77^	EAAT2	SD rats	Male	270–290	10/10	mention	not mention	NOT SAME	MA	LI4,LR3	mention	30 min	21	35d	Riluzole	OFT:NC; W:FW; SI:RSI.
Xu 2016^79^	CUMS & Solitary	SD rats	Male	200 ± 20	10/10	mention	not mention	SAME	EA	GV20,GV29	mention	30 min	28	28d	Fluoxetine	OFT:NC,NR;
Xu 2016#^80^	CUMS & Solitary	SD rats	Male	180 ± 20	8/8	mention	not mention	SAME	MA	GV20,GV29	mention	10 min	21	21d	Fluoxetine	OFT:NC,NR;W:FW; SI:CSI.
Yang 2013^81^	CUMS & Solitary	SD rats	Male	200 ± 20	10/10	mention	not mention	SAME	EA	ST36	mention	20 min	21	21d	Fluoxetine	OFT:NC,NR; W:GW; SI:CSI.
Yang 2013#^82^	CUMS & Solitary	SD rats	Male	200–220	8/8	mention	not mention	NOT SAME	EA	GV20,GV29	mention	30 min	18	21d	Citalopram	OFT:NC,NR;W:FW; SI:RSI.
Yu 2006^83^	CUMS	SD rats	Male	120–150	8/8	mention	not mention	NOT SAME	EA	GV20,GB34	mention	30 min	7	14d	Clomipramine	OFT:NC,NR.
Yu 2012^84^	CUMS & Solitary	SD rats	Male	160–180	10/10	mention	not mention	SAME	EA	GV20,GV29	mention	not mention	21	21d	Fluoxetine	OFT:NC,NR; W:FW; SI:CSI.
Yu 2016^85^	CUMS & Solitary	SD rats	Male	180 ± 10	8/8	mention	not mention	SAME	MA	GV20,GV29	mention	10 min	21	21d	Fluoxetine	OFT:NC,NR;W:FW; SI:CSI.
Yu 2006#^86^	CUMS	SD rats	Male	200–250	10/10	not mention	not mention	NOT SAME	EA	GV20, Anmian	mention	40 min	18	42d	Chlorimipramine	W:FW; SI:CSI,RSI.
Zhang 2005^87^	CUMS	SD rats	Male	180–220	10/10	mention	not mention	NOT SAME	MA	GV20,PC6,GV24,SP6	mention	10 min	21	21d	Fluoxetine	OFT:NC,NR; W:FW; SI:CSI.
Zhang 2008^88^	CUMS & Solitary	SD rats	Male	160–200	10/10	mention	not mention	SAME	EA	GV20,GV29	mention	30 min	21	21d	Fluoxetine	OFT:NC,NR; SI:CSI.
Zhang 2016^89^	CUMS	SD rats	Male	180–200	10/10	mention	not mention	SAME	MA	GV20,GV29	mention	10 min	21	21d	Fluoxetine	OFT:NC,NR;SI:CSI.
Zhang# 2016^90^	CUMS	SD rats	Male/Female	220–290	20/20	mention	not mention	SAME	MA	GV20,PC6,SP6	mention	30 min	21	21d	Fluoxetine	OFT:NC,NR;W:FW; SI:CSI.
Zhang 2016&^91^	CUMS & Solitary	SD rats	Male	220–270	12/12	mention	not mention	NOT SAME	EA	LI4,LR3	mention	30 min	14	21d	Riluzole	OFT:NC,NR;W:FW; SI:RSI.
Zhang 2016*^92^	CUMS & Solitary	SD rats	Male	220–240	10/10	mention	not mention	SAME	MA	GV20,GV29	mention	10 min	28	28d	Fluoxetine	W:FW; SI:RSI.
Zhao 2005^93^	CUMS & Solitary	SD rats	Male	200–230	8/8	mention	not mention	SAME	EA	GV20,SP6	mention	20 min	21	21d	Fluoxetine	OFT:NC,NR; W:GW.
Zheng 2013^94^	CUMS	Wistar rats	Male	180–220	16/16	mention	not mention	NOT SAME	EA	GV20,GV24,EX-HNl	mention	20 min	14	14d	Fluoxetine	OFT:NC
Zhou 2008^95^	PSA	Wistar rats	Male	200 ± 20	10/10	mention	not mention	NOT SAME	MA	GV20,PC6,GV24,SP6	mention	10 min	14	14d	Doxepin	OFT:NC,NR.
Zhu 2015^96^	CUMS	SD rats	Male	not mention	9/9	mention	not mention	SAME	EA	GV20, Anmian	mention	30 min	14	28d	Chlorimipramine	OFT:NC,NR;SI:RSI.
Zhuang 2007^97^	CUMS & Solitary	SD rats	Female/Male	220–290	12/12	mention	not mention	SAME	MA	GV20,BL15,BL18	mention	15 min	21	21d	Fluoxetine	OFT:NC,NR; W:FW; SI:CSI.

Notes: CFSS: chronic forced swim stress model; CRS: chronic restraint stress model; CUMS: chronic unpredictable mild stress model; CSI:capacity of sucrose intake; EA:electroacupuncture; EAAT1 model:lateral ventricle injection excitatory amino acid transporter 1 antagonists model; EAAT2 model: prefrontal cortex injection excitatory amino acid transporter 2 antagonists model; FW: final weight; GW:gain weight; IA model:intracerebroventricular injection of ibotenic acid model; L-AAA:lateral ventricle injection L-α-aminoadipic acid model; LH: learned helpless model; MA: manual acupuncture; NC: the number of crossings; NOT SAME: intervention after or in the process of modeling. NR: the number of rearings; OFT:open field test; PSA: pre-shocked animals; RSI:rate of sucrose intake; SAME:intervention and modeling at the same time;SI:sucrose intake; W:weight;

### Study quality and publication bias

The score of each study ranged from 2 to 7 out of a total 10 points quality checklist. Eleven studies got 2 points, forty-one studies got 3 points, twenty-nine studies got 4 points, eight studies got 5 points, one study got 6 points, two studies got 7 points (Table 2). Twenty-four studies utilized anesthetics which have no effect on depressive symptoms. Forty-four studies described the control of temperature, including control of the room and rats’ anal temperature. Fifty-six studies were published in peer-reviewed journals. None of the studies described the sample size calculation, allocation concealment, blinded assessment of outcome. Twelve studies described compliance with animal welfare regulations and three studies declared potential conflicts of interest. Only two studies did not describe randomization.

**Table 2:** Assessment of the quality of studies included.

Research	(1)	(2)	(3)	(4)	(5)	(6)	(7)	(8)	(9)	(10)	Total
Fan 2016*^24^	√	×	√	×	×	√	√	√	√	√	7
Zhang 2016^89^	√	×	√	×	×	√	√	√	√	√	7
Duan 2016#^20^	√	×	√	×	×	×	√	√	√	√	6
Duan 2016^19^	√	×	√	×	×	×	√	√	√	×	5
Jing 2016^36^	√	×	√	×	×	√	√	×	√	×	5
Lu 2016#^50^	√	×	√	×	×	×	√	√	√	×	5
Mo 2014^53^	√	×	√	×	×	√	√	×	√	×	5
Shi 2015^58^	√	×	√	×	×	×	√	√	√	×	5
Sun 2016^66^	√	×	√	×	×	√	√	×	√	×	5
Tang 2013^67^	√	×	√	×	×	√	√	×	√	×	5
Wu 2007^76^	√	×	√	×	×	√	√	×	√	×	5
Cheng 2015^14^	√	×	√	×	×	√	√	×	×	×	4
Dai 2010^15^	√	×	√	×	×	√	×	×	√	×	4
Deng 2013^16^	√	×	√	×	×	√	√	×	×	×	4
Ding 2016^17^	√	×	√	×	×	×	√	×	√	×	4
Duan 2008^18^	√	×	√	×	×	√	×	×	√	×	4
Hu 2013^27^	√	×	√	×	×	√	√	×	×	×	4
Huang 2005^29^	√	×	√	×	×	√	√	×	×	×	4
Li 2008^38^	√	×	√	×	×	√	×	×	√	×	4
Li 2014^41^	√	×	√	×	×	×	√	×	√	×	4
Liu 2009^45^	√	×	√	×	×	×	√	×	√	×	4
Liu 2012^46^	√	×	√	×	×	×	√	×	√	×	4
Lu 2008^47^	√	×	√	×	×	√	×	×	√	×	4
Lu 2013^48^	√	×	√	×	×	×	×	√	√	×	4
Luo 2016^51^	√	×	√	×	×	√	×	×	√	×	4
Ma 2016^52^	√	×	√	×	×	×	√	×	√	×	4
Qin 2010^55^	√	×	√	×	×	×	√	×	√	×	4
Song 2016^61^	√	×	√	×	×	×	√	×	√	×	4
Song 2014#^62^	√	×	√	×	×	√	×	×	√	×	4
Sun 2013^64^	√	×	√	×	×	√	×	×	√	×	4
Sun 2014^65^	√	×	√	×	×	×	√	×	√	×	4
Tang 2014^68^	√	×	√	×	×	×	√	×	√	×	4
Wang 2016^75^	√	×	√	×	×	√	×	×	√	×	4
Xiao 2014&^78^	√	×	√	×	×	√	×	×	√	×	4
Xu 2016^79^	√	×	√	×	×	×	×	√	√	×	4
Yang 2013#^82^	√	×	√	×	×	×	×	√	√	×	4
Yu 2006#^86^	√	×	×	×	×	×	√	√	√	×	4
Zhang 2016&^91^	√	×	√	×	×	√	√	×	×	×	4
Zhang 2016*^92^	√	×	√	×	×	√	√	×	×	×	4
Zhu 2015^96^	√	×	√	×	×	×	√	×	√	×	4
Bao 2014^11^	√	×	√	×	×	×	×	×	√	×	3
Bao 2014#^12^	√	×	√	×	×	×	×	×	√	×	3
Chen 2011^13^	√	×	√	×	×	×	×	×	√	×	3
Fan 2016^22^	√	×	√	×	×	×	×	×	√	×	3
Fan 2016#^23^	√	×	√	×	×	×	×	×	√	×	3
Guo 2016^26^	√	×	√	×	×	×	√	×	×	×	3
Hu 2014^28^	√	×	√	×	×	×	√	×	×	×	3
Ji 2013^30^	√	×	√	×	×	×	×	×	√	×	3
Jia 2005^31^	√	×	√	×	×	×	√	×	×	×	3
Jiang 2007^32^	√	×	√	×	×	√	×	×	×	×	3
Jiao 2008^34^	√	×	√	×	×	×	×	×	√	×	3
Jin 2015^35^	√	×	√	×	×	×	√	×	×	×	3
Li 2007^37^	√	×	√	×	×	×	×	×	√	×	3
Li 2011^39^	√	×	√	×	×	×	√	×	×	×	3
Li 2011#^39^	√	×	√	×	×	×	√	×	×	×	3
Li 2011&^40^	√	×	√	×	×	×	×	×	√	×	3
Liu 2005^43^	√	×	√	×	×	×	×	×	√	×	3
Liu 2008^44^	√	×	√	×	×	×	×	√	×	×	3
Lu 2016^49^	√	×	√	×	×	×	×	√	×	×	3
Pan 2016^54^	√	×	√	×	×	×	√	×	×	×	3
Shao 2016^56^	√	×	√	×	×	×	√	×	×	×	3
Shi 2007^57^	√	×	√	×	×	×	×	×	√	×	3
Song 2015^60^	√	×	√	×	×	×	√	×	×	×	3
Sun 2003^63^	√	×	√	×	×	×	×	×	√	×	3
Teng 2013^69^	√	×	√	×	×	×	√	×	×	×	3
Wang 2008^70^	√	×	√	×	×	×	×	×	√	×	3
Wang 2013^73^	√	×	√	×	×	×	×	×	√	×	3
Wang 2014^74^	√	×	√	×	×	×	×	×	√	×	3
Xiao 2014#^77^	√	×	√	×	×	√	×	×	×	×	3
Xu 2016#^80^	√	×	√	×	×	×	√	×	×	×	3
Yang 2013^81^	√	×	√	×	×	×	×	×	√	×	3
Yu 2006^83^	√	×	√	×	×	×	×	×	√	×	3
Yu 2012^84^	√	×	√	×	×	×	√	×	×	×	3
Yu 2016^85^	√	×	√	×	×	×	√	×	×	×	3
Zhang 2005^87^	√	×	√	×	×	×	×	×	√	×	3
Zhang 2008^88^	√	×	√	×	×	×	√	×	×	×	3
Zhang# 2016^90^	√	×	√	×	×	×	√	×	×	×	3
Zheng 2013^94^	√	×	√	×	×	×	×	×	√	×	3
Zhou 2008^95^	√	×	√	×	×	×	×	×	√	×	3
Zhuang 2007^97^	√	×	√	×	×	×	×	×	√	×	3
Fan 2013^21^	√	×	√	×	×	×	×	×	×	×	2
Fu 2008^25^	√	×	√	×	×	×	×	×	×	×	2
Jiang 2013^33^	√	×	√	×	×	×	×	×	×	×	2
Lin 2008^42^	√	×	√	×	×	×	×	×	×	×	2
Song 2014^59^	√	×	√	×	×	×	×	×	×	×	2
Wang 2009^71^	√	×	√	×	×	×	×	×	×	×	2
Wang 2010^72^	√	×	×	×	×	×	×	×	√	×	2
Xiao 2014^77^	√	×	√	×	×	×	×	×	×	×	2
Xiao 2014*^77^	√	×	√	×	×	×	×	×	×	×	2
Zhao 2005^93^	√	×	√	×	×	×	×	×	×	×	2

Notes: (1) sample size calculation; (2) randomization to treatment group; (3) allocation concealment; (4) blinded assessment of outcome; (5) correctness of methods of modeling; (6) avoidance of anesthetics with resistance to depressive; (7)statements describing control of temperature; (8) compliance with animal welfare regulations; (9) publication in a peer-reviewed journal; (10) declared any potential conflict of interest.

### Outcome and effect estimates

Based on various behavior indicators of the included studies, different pooled data of 69 studies in 67 articles were applied in meta-analysis and 21 studies in 20 articles were not applied in meta-analysis because they did not provide raw data. The data lacked details like observing time or specifications were also not pooled in the analysis because it indicated that the data is not precise enough. All continuous data were presented as mean ± SD.

**Figure 2:**
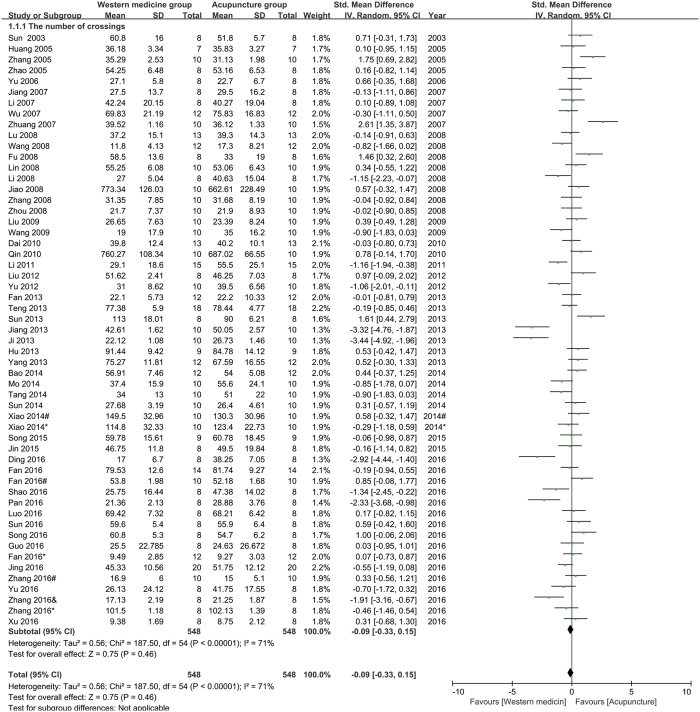
Pool result of acupuncture versus western medicine on NC.

The number of crossings (NC) in open field test (OFT): fifty-five studies were pooled in the meta-analysis of NC. The result (Fig. 2) showed that acupuncture had not less effect than western medicine on improving NC in depression model rats (n = 1096, SMD = −0.09, 95%CI = [−0.33 to 0.15], P = 0.46; heterogeneity: Chi^2^ = 187.50, df = 54, P < 0.00001, I^2^ = 71%). Ten studies reported NC was not pooled in the meta-analysis for the lack of observing time or specification.

**Figure 3:**
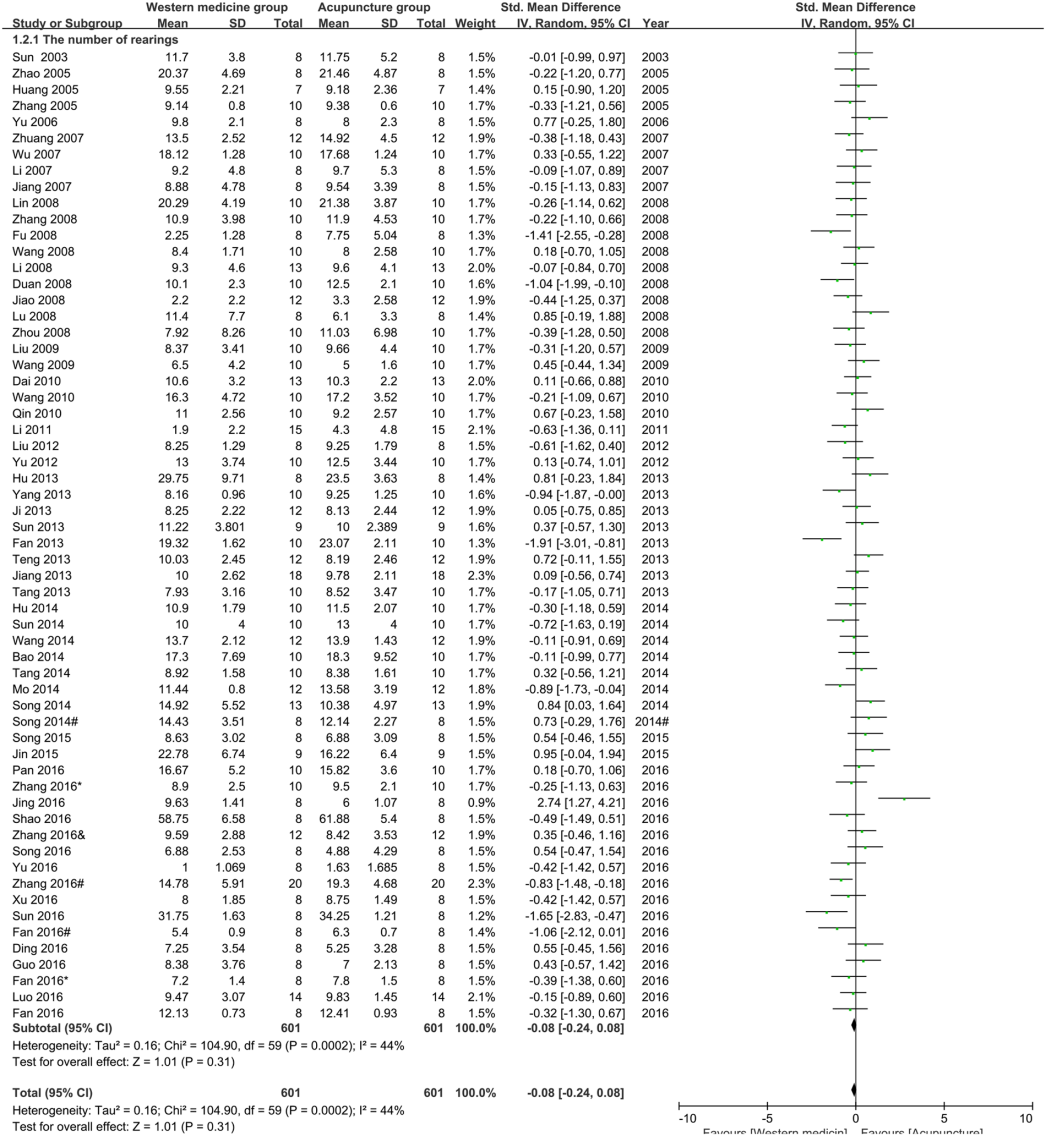
Pool result of acupuncture versus western medicine on NR.

The number of rearings (NR) in open field test (OFT): sixty studies were pooled in the meta-analysis of NR. The result (Fig. 3) showed that acupuncture had not less effect than western medicine on improving NR in depression model rats (n = 1202, SMD = −0.08, 95%CI = [−0.24 to 0.08], P = 0.0002; heterogeneity: Chi^2^ = 104.90, df = 59, P = 0.0002, I^2^ = 44%). Two studies reported NR were not pooled in the meta-analysis for the lack of observing time.

**Figure 4:**
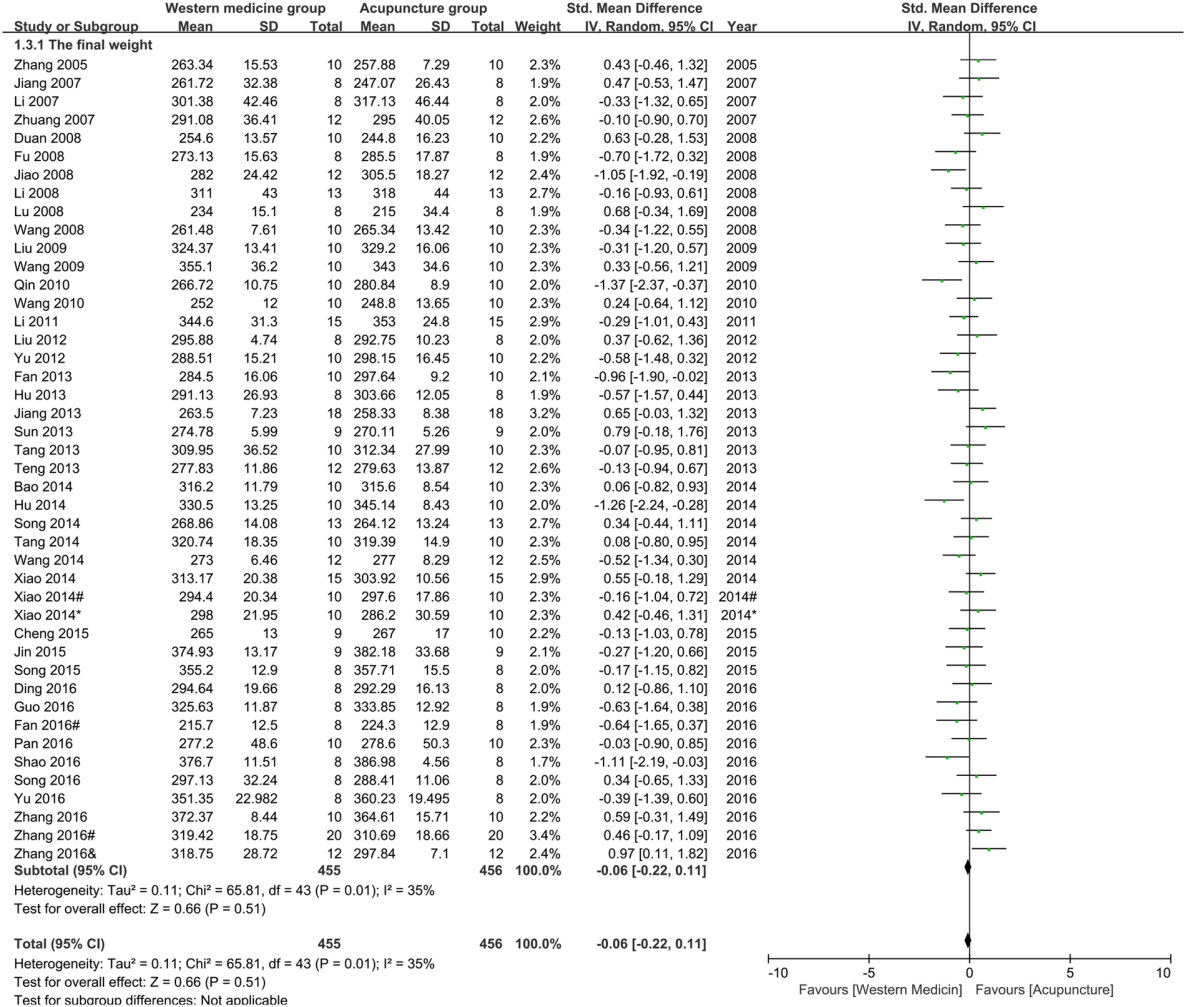
Pool result of acupuncture versus western medicine on FW.

Final weight (FW): forty-four studies were pooled in the meta-analysis of FW. The result (Fig. 4) showed that acupuncture had not less effect than western medicine on improving FW of depression model rats (n = 911, SMD = −0.06, 95%CI = [−0.22 to 0.11], P = 0.51; heterogeneity: Chi^2^ = 65.81, df = 43, P = 0.01, I^2^ = 35%).

**Figure 5:**
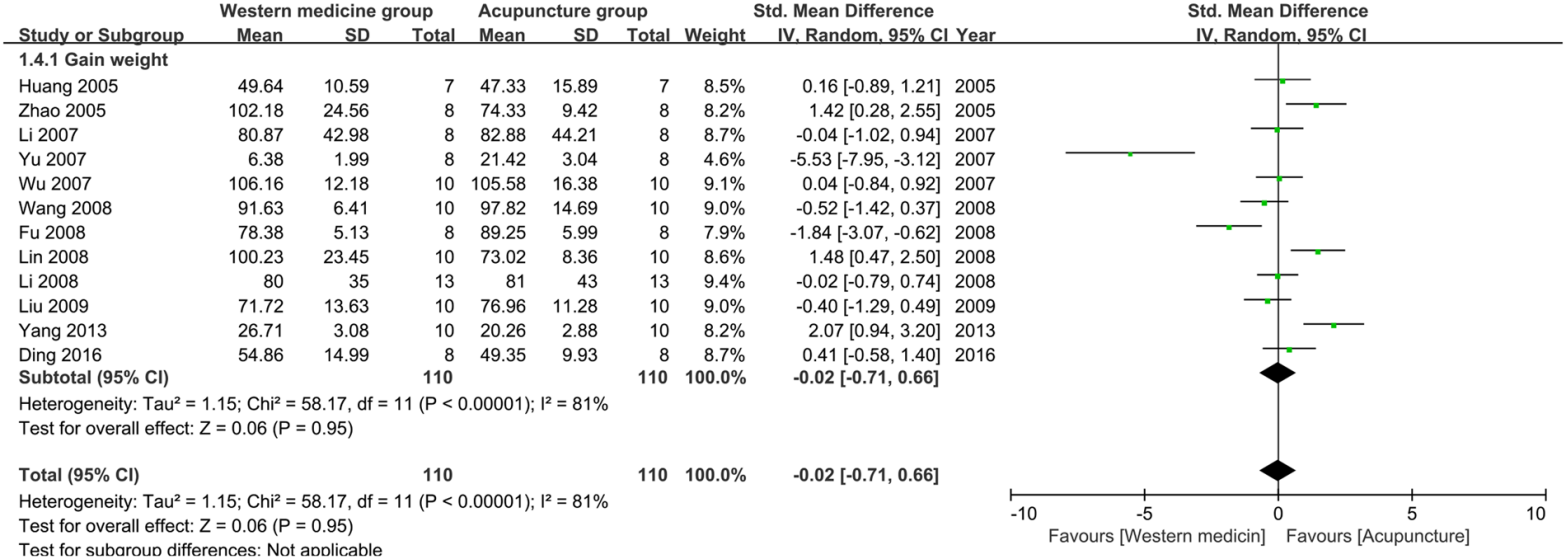
Pool result of acupuncture versus western medicine on GW.

Gain weight (GW): twelve studies were pooled in the meta-analysis of GW. The result (Fig. 5) showed that acupuncture had not less effect than western medicine on improving GW of depression model rats (n = 220, SMD = −0.02, 95%CI = [−0.71 to 0.66], P = 0.95; heterogeneity: Chi^2^ = 58.17, df = 11, P < 0.00001, I^2^ = 81%).

**Figure 6:**
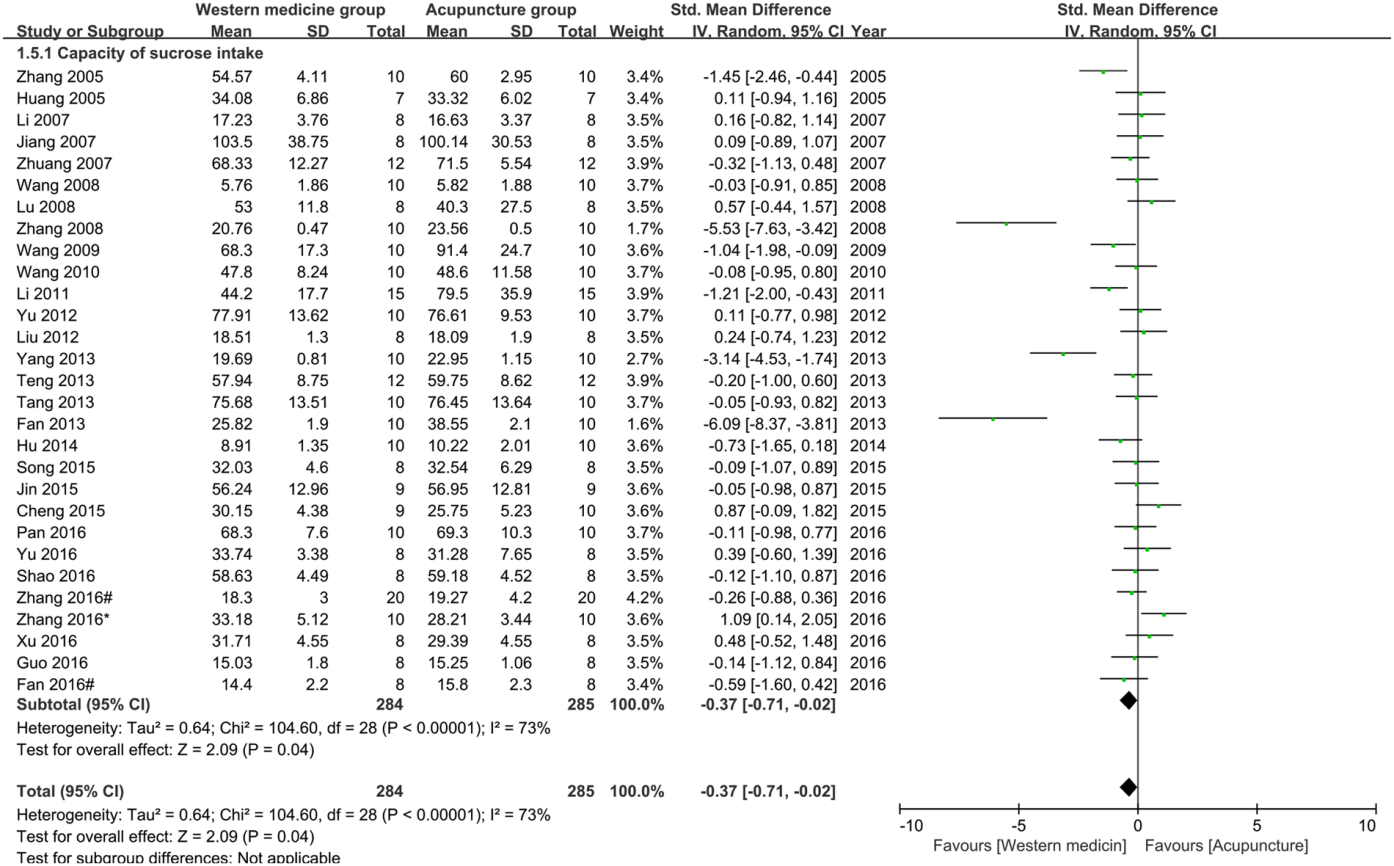
Pool result of acupuncture versus western medicine on CSI.

The capacity of sucrose intake (CSI): twenty-nine studies were pooled in the meta-analysis of CSI. The result (Fig. 6) showed that acupuncture had poorer effects than western medicine on improving CSI of depression model rats (n = 568, SMD = −0.37, 95%CI = [−0.71 to −0.02], P = 0.04; heterogeneity: Chi^2^ = 104.60, df = 28, P < 0.00001, I^2^ = 73%). Five studies reported CSI were not pooled in the meta-analysis because of lacking specification.

**Figure 7:**
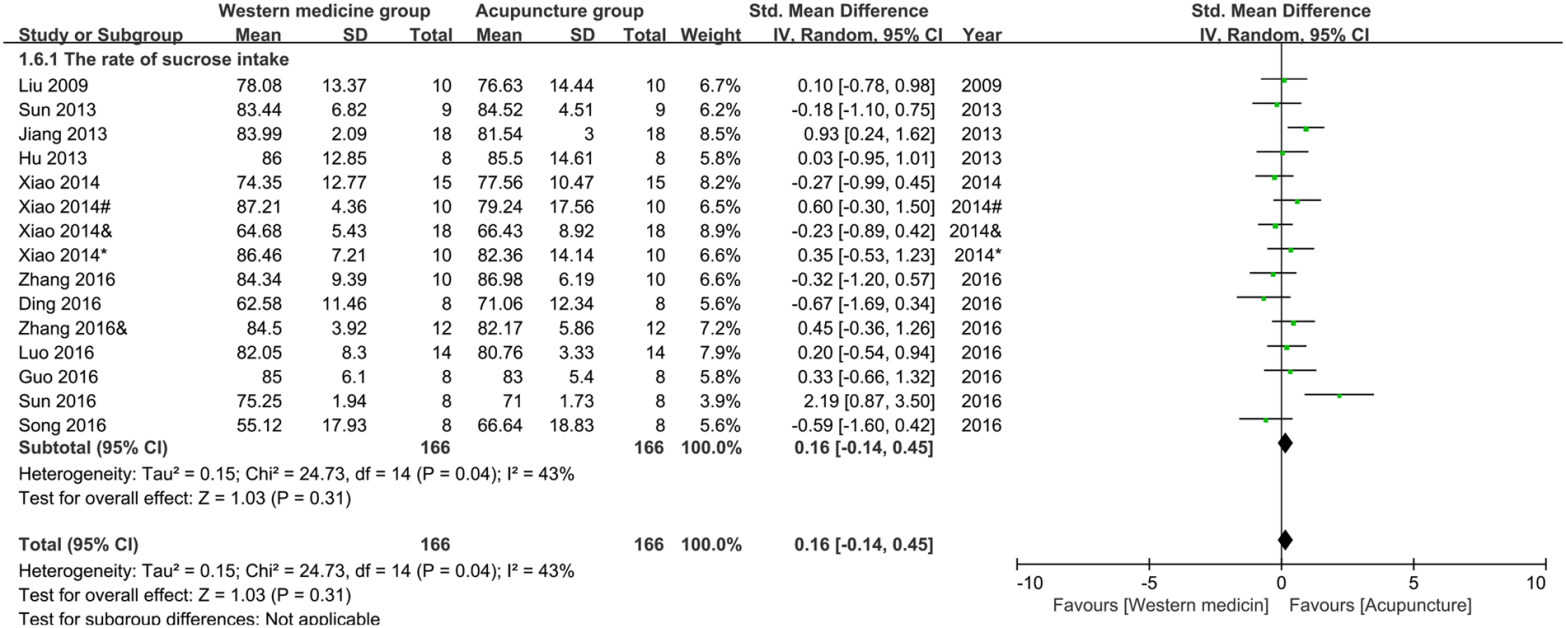
Pool result of acupuncture versus western medicine on RSI.

The rate of sucrose intake (RSI): fifteen studies were pooled in the meta-analysis of RSI. The result (Fig. 7) showed that acupuncture had not less effect than western medicine on improving RSI of depression model rats (n = 332, SMD = 0.16, 95%CI = [−0.14 to 0.45], P = 0.31; heterogeneity: Chi^2^ = 24.73, df = 14, P = 0.04, I^2^ = 43%).

### Proposed mechanisms

Seventy-six included studies provided detailed descriptions about possible mechanisms of acupuncture in behavior changes of depression models. A summary of proposed mechanisms is shown in Table 3.

**Table 3:** Proposed mechanisms of acupuncture in behavior changes in depression models of studies included.

Research	Proposed mechanisms
Bao 2014^11^	Increased 5-HT in serum.
Bao 2014#^12^	Relieved impairment of hippocampal neurons.
Chen 2011^13^	Increased expression of Bcl-2 in hippocampus.
	Decreased expression of Bax in hippocampus.
Cheng 2015^14^	Regulated the expression of IL-6R in frontal cortex and hippocampus.
Dai 2010^15^	Down-regulated level of phospho-JNK in hippocampus.
	Reduced hippocampal apoptotic rate.
Deng 2013^16^	Decreased expressions of IL-1β, IL-6 in hippocampus and serum.
Duan 2008^18^	Increased p-CREB in hippocampus.
Duan 2016^19^	Increased 5-HT, Glu, GABA in hippocampus.
	Increased levels of TrkB, BDNF, p-CREB,PKA and decreased CaMKII in the hippocampus.
	Up-regulated expression levels of PKA mRNA and TrkB mRNA and down-regulated expression levels of CaMKII mRNA in the hippocampus.
Duan 2016#^20^	Increased 5-HT in hippocampus.
	Increased 5-HIAA level.
	Up-regulated levels of TPH and 5-HT1A.
	Up-regulated expression levels of TPH mRNA and 5-HT1A mRNA.
Fan 2013^21^	Decreased IL-2,IL-6 and NO in serum.
Fan 2016^22^	Regulated levels of 5-HT, NE, BDNF and expression of Bax/Bcl-2.
Fan 2016#^23^	Down-regulated expressions of SNAP25, VAMP1, VAMP2, VAMP7 and syntaxin1.
Fan 2016*^24^	Down-regulated expressions of SNAP25, VAMP1, VAMP2, VAMP7 and syntaxin1.
Fu 2008^25^	Up-regulated expressions of CREB, BDNF in hippocampus.
Guo 2016^26^	Decreased level of ROS in hippocampus.
	Decreased protein expression levels of cytochrome C,caspase-3, and AIF in hippocampus
Hu 2013^27^	Decreased IL-1β,IL-6,TNF-α in serum, hippocampus and frontal cortex.
Hu 2014^28^	Down-regulated expressions of RAGE, Activin A, CNTF Rα, EGFR, E-Selectin, and Resistin in hippocampus.
	Decreased IL-1β, IL-10 in hippocampus.
Huang 2005^29^	Decreased CORT,ACTH in serum.
Ji 2013^30^	Increased the GFAP and astrocyte in hippocampus.
Jia 2005^31^	Decreased IL-1, CRF in hypothalamus.
	Decreased CRF in hypophysis.
	Decreased ACTH, CORT in adrenal gland.
	Down-regulated expressions of CRF_1_mRNA, POMC mRNA in hypophysis.
	Up-regulated expressions of GRmRNA in hippocampus, hypothalamus, hypophysis and MRmRNA, 5-HT_1A_mRNA in hippocampus.
Jiang 2007^32^	Incresed cAMP in hippocampus.
	Up-regulated expressions of CREB,BDNF in hippocampus.
Jiang 2013^33^	Repaired astrocytes in CA1, DG area in hippocampus.
	Increased GFAP, GFAPmRNA in hippocampus.
Jin 2015^35^	Down-regulated expression of NF-kB, COX-2, and COX-2 mRN in hippocampus and decreased PGE2 in hippocampus.
Jing 2016^36^	Down-regulated the expression of PDE4A and PDE4D mRNA in hippocampus.
Li 2007^37^	Increased cAMP in hippocampus.
Li 2008^38^	Increased bcl-2 in hippocampus.
	Reduced hippocampal apoptotic rate.
Li 2011^39^	Regulated the gene expression of Dnmt3L and MBP in hippocampus.
Li 2011&^40^	Increased β-EP in serum and µ receptors level in hypothalamus.
Li 2014^41^	Increased 5-HT and 5-HTT in hippocampus.
Lin 2008^42^	Increased BDNF in the hippocampal. Dncreased TGF-β1 in serum.
Liu 2008^44^	Regulated GFAP,BDNF,GDNF in hippocampus.
Liu 2009^45^	Increased 5-HT,DA,NE in hippocampus.
Liu 2012^46^	Incresed AC transformation ratio,cAMP level, and PKA activity.
Lu 2008^47^	Reduced CORT in serum.
	Increased expressions of PKA and PKC in hippocampus.
Lu 2013^48^	Regulated levels of ERK1/2,p-ERK1/2, CREB, p-CREB in the hippocampus.
Lu 2016^49^	Reduced levels of NO, PGE2, iNOS and COX-2 in the hippocampus and prefrontal cortex.
	Inhibited the activation of NF-kB in the hippocampus and prefrontal cortex.
Lu 2016#^50^	Increased the mRNA and protein expression of IL-1β, IL-6, and TNF-α in the hippocampus and prefrontal cortex and cytokine concentrations in serum.
Luo 2016^51^	Regulated GS,EAAT1,EAAT2 of astrocyte cell in prefrontal cortex.
Ma 2016^52^	Upregulated expression of TRH and TRH mRNA in the hypothalamus.
Mo 2014^53^	Upregulated expression of TRH and TRH mRNA in the hypothalamus.
Pan 2016^54^	Regulated Glu, GABA in hippocampal
	Increased DA,5-HT in serum.
	Regulated expressions of GAD65, EATT3,GAT1, NR2B in hippocampal.
Qin 2010^55^	Increased NT in hypothalamus and ileum.
Shao 2016^56^	Downregulated expressions of NF-kB, NO, iNOS.
Shi 2007^57^	Reduced levels of CORT,ACTH,CRH.
Shi 2015^58^	Increased 5-HT in hippocampal and BDNF in serum levels,and decreased CORT in adrenal.
Song 2014^59^	Increased the production of MT.
	Regulated ET, NO, ET-1 in serum.
	Regulated leves of renin Ang II (Angiotensin II), AT1R (Angiotensin II Type 1 Receptor), ACE (Angiotensin converting enzyme).
	Regulated MOD, SOD in serum.
Song 2015^60^	Regulated BDNF, bcl-2, CREB,and EPK signaling pathway.
Song 2016^61^	Reduced CORT,ACTH,CRH in serum.
Song 2014#^62^	Down-regulated expression of AngII, AT1R and ACE I in the arterial tissue.
Sun 2013^64^	Promoted repair of astrocytes.
Sun 2014^65^	Down-regulated expression of p-JNK, c-jun, Caspase-3.
Sun 2016^66^	Decreased CORT, ACTH in serum.
Tang 2013^67^	Increased 5-HT,DA,NE in hippocampus.
Tang 2014^68^	Increased 5-HT,DA,NE in hippocampus and loubus fromatis.
Teng 2013^69^	Increased GAS and reduced NPY,CGRP in colonic mucosa,increased GAS,NPY,CGRP in hypothalamus.
	Increased β-EP in hypothalamus and reduced β-EP in colonic mucosa.
Wang 2009^71^	Incresed expression of 5-HT2A receptor mRNA in hippocampus.
	Reduced expression of 5-HT1A receptor mRNA in hippocampus.
Wang 2010^72^	Increased BDNF in hippocampus.
Wang 2013^73^	Regulated β-EP in serum and brain tissue.
Wang 2016^75^	Promoted repair of hippocampus CA3 region.
Wu 2007^76^	Repaired the lesion of multiple organs.
	Promoted 5-HT synthesis.
Xiao 2014^77^	Improved expression of GS mRNA, EAAT1 mRNA,EAAT2 mRNA.
Xiao 2014&^78^	Improved the injury of hippocampal astrocytes.
Xu 2016^79^	Up-regulated level of TGF-3β in hippocampal and down-regulated expression of bFGF.
Xu 2016#^80^	Regulated levels of ERK1/2, p-ERK1/2, CREB, p-CREB and BDNF in the hippocampus.
Yang 2013#^82^	Regulated levels of TrkB and BDNF.
Yu 2012^84^	Increased 5-HT,DA,NE in brain.
Yu 2016^85^	Regulated levels of PKA,CREB in prefrontal cortex and IL-6,TNF-α in serum.
Zhang 2008^88^	Reduced CORT and CRF.
	Improved expression of GR,NMDA,NR2B, PKA, CREB, Nestin in hippocampus.
Zhang 2016^89^	Regulated levels of ERK1/2,p-ERK1/2, CREB, p-CREB and BDNF in the hippocampus.
Zhang# 2016^90^	Regulated Glu,GABA in hippocampus.
	Down-regulated expressions of NMDA-receptor subunits NRl and NR2A in hippocampus.
Zhang 2016&^91^	Increased GFAP,GS,Glu-C4,Gln-C4,GABA-C2 in prefrontal cortex and hippocampus.
Zhang 2016*^92^	Regulated BDNF, acH3K9, HDAC2 and PKA signaling pathway.
Zhao 2005^93^	Reduced CORT,ACTH in serum.
	Increased 5-HT, NE in brain.
Zheng 2013^94^	Down-regulated expression of CRF in hypothalamus.

Notes: β-EP, β-endorphin; p-CREB, phosp-horylated cAMP response element binding protein; 5-HT, 5-hydroxytryptamine; 5-HT1A: 5-hydroxyindoleacetic acid; 5-HTT, 5-hydroxytryptamine transporter; ACEI, Angiotensin converting enzyme I; acH3K9, acetylation of histone 3 lysine 9; ACTH, adrenocorticotropic hormone; AIF, apotposis inducing factor; AngII, angiotensin II; AT1R, angiotensin II Type 1 Receptor; Bax, Bcl-2 Assaciated X protein; Bcl-2, B-cell lymphoma 2; BDNF, brain derived neurotrophic factor; bFGF, basic fibroblast growth factor; CaMKII, calcium-calmodulin-dependent protein kinase II; cAMP, adenosine 3’, 5’-cyclic monophos-phate; CGRP, calcitonin generelated peptide; CNTF Rα, ciliary neurotrophic factor receptor α; CORT, corticoserone; COX-2, cyclo-oxygenase-2; CRF, corticotropin releasing factor; CRH, corticotropin releasing hormone; DA, Dopi Amine; Dnmt, DNA methytransferase; EAAT1, excitatory amino acid transporter 1; EAAT2, excitatory amino acid transporter 2; EGFR, epithelial growth factor receptor; ERK, extracellular signal-regulated kinase; GABA, γ-aminobutyric acid; GAS, Gastrin; GDNF, glial cell line-derived neurotrophic factor. GFAP, glial fibrillary acidic protein; Gln, glutamine; Glu, glutamate; GR, glucocorticoid receptor; GS, glutamine synthetase; HDAC2, histone deacetylase 2; HPA, hypothalamus-pituitary-adrenal; IL-1β, Interleukin-1β; IL-6, interleukin-6; IL-6r, Interleukin-6r; IL-10, Interleukin-10; iNOS, inducible Nitric Oxide Synchase; JNK, c-Jun N-terminal kinasesignal; MBP: myelin basic protein; MLT, motilin; MOD, Malondialdehyde; MT, melatonin; NE, norepinephrine; Nestin, neuroepithelial stem protein; NF-kB, nuclear factor kappa B; NMDA, N-methy-D-aspartate; NO, nitric oxide; NPY, neuropeptide-y; NR2B, NMDA receptor subunits 2B; NT, neurotensin; PDE4, phosphodiesterase 4; PGE2, prostaglandin E2; PKA, protein kinase A; PKC, protein kinase C; RAGE, receptor for advanced glycation end-products; RAS, renin-angiotensin system; ROS, reactive oxygen species; SNAP, synaptic soluble Nethylmaleimide-sensitive factor attachment receptor; SOD, Superoxide dismutase; SS, somatostatin; TGF-3β, transforming growth factor beta 3; TNF-α, tumor necrosis factor-α; TPH, tryptophan hydroxylase; TRH,Thyrotropin releasing hormone; TrkB, tropomyosin receptor kinase B; VAMP, vesicle-associated membrane protein.

### Investigation of heterogeneity

Sensitivity analysis: sensitivity analysis showed that heterogeneity reduced to be acceptable after removing two studies, three studies and one study of which coincidence degree was poor among other studies pooled in the meta-analysis of NR, CSI and RSI, respectively. So, to a large extent, these studies may be the origination of heterogeneity in NR, CSI and RSI. Tests for overall effects in these cases showed that acupuncture had not less effect than western medicine on improving NR (Z = 1.14 (P = 0.25)), CSI (Z = 1.00 (P = 0.32)), RSI (Z = 0.68 (P = 0.50)) in depression model rats. The heterogeneity was not substantially altered after dismissing any study pooled in the meta-analysis of NC and GW.

Subgroup analysis: to further investigate the source of heterogeneity among the studies pooled in the meta-analysis of NC and GW, a subgroup analysis was conducted. Study characteristics, including different types of acupuncture (acupuncture or electroacupuncture), different stimulation acupoints (scalp acupoints, body acupoints, or scalp acupoints and body acupoints), different intervention time (modeling and intervention at the same time or intervention after or in the process of modeling) and different duration of treatment (more than 21d or less than 14d) are thought to be potential factors affecting the effect of acupuncture. The subgroup analysis was done on the basis of these characteristics. It’s failed to subgroup the results by acupuncture method and duration on the basis of GW, since only one study selected manual acupuncture and all the studies’ duration were more than 21d. Unfortunately, we did not find the exact source of heterogeneity among studies by subgroup analysis.

Sensitivity analysis and subgroup analysis were not developed because heterogeneity among studies that were pooled in the meta-analysis of FW (I^2^ = 35% (P = 0.01)) is acceptable. Details of sensitivity analysis and subgroup analysis were shown in Table 4 and the raw data were shown in Figs S1–S9.

**Table 4:** Details of sensitivity analysis and subgroup analysis.

Sensitivity analysis	Removed studies	Changes of I^2^(P)
NR	Jing2016^36^, Fan2013^21^	44% (0.0002) → 29% (0.02)
CSI	Zhang2008^88^, Fan2013^21^,Yang2013^81^	73% (0.04) → 32% (0.06)
RSI	Sun2016^66^	43% (0.04) → 14% (0.30)
Subgroup analysis	Factors	I^2^ in Subgroups
NC	different types of acupuncture	EA: 69%, MA:76%
	different stimulation acupoints	SA:55%, BA:74%, SA+BA:78%
	different intervention time	SAME:74%, NOT SAME:66%
	different duration of treatment	More than 21d:72%, Less than 14d:41%
GW	different stimulation acupoints	SA:89%, BA:86%, SA+BA:73%
	different intervention time	SAME:96%, NOT SAME:0%

Notes: CSI:capacity of sucrose intake; EA:electroacupuncture; GW:gain weight; MA: manual acupuncture; NC: the number of crossings; NOT SAME: intervention after or in the process of modeling. NR: the number of rearings; RSI:rate of sucrose intake; SA:scalp acuponits; BA:body acupoints; SAME:intervention and modeling at the same time.

### Assessment of publication bias

**Figure 8:**
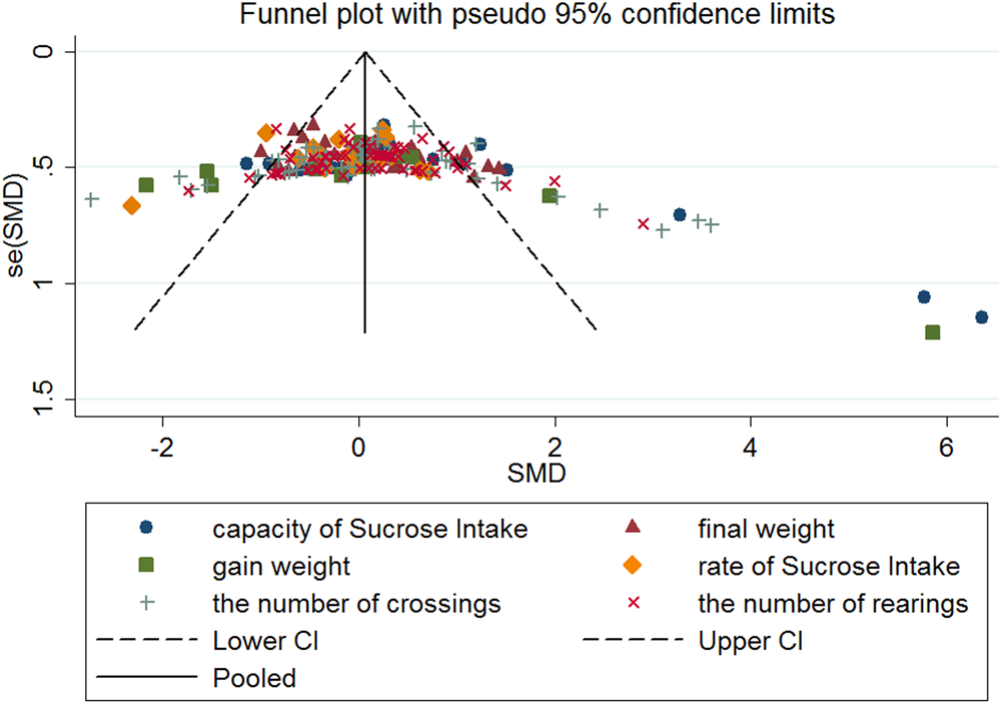
The funnel plot of the effects of acupuncture on behavioral changes in experimental depression.

Funnel plot showed asymmetry and it indicated a potential publication bias (Fig. 8). Begg’s test showed there was no significant publication bias (p = 0.548), but the Egger’s test (p < 0.001) indicated publication bias possibly existed (Fig. S10). In addition, all of the studies published by Chinese authors and 47 studies published in Chinese journals^11–13, 15, 17, 18, 22, 23, 30, 34, 36–38, 40, 41, 43, 45, 47, 51–53, 55, 57, 58, 61–68, 70, 72–76, 78, 79, 81, 83, 87, 94–97^, 10 studies published in English journals (9 of them came from SCI source journals)^19, 20, 24, 46, 48–50, 82, 86, 89^, 33 studies came from PhD/MD. Thesis in China^14, 16, 21, 25–29, 31–33, 35, 39, 42, 44, 54, 56, 59, 60, 69, 71, 77, 80, 84, 85, 88, 90–93^.

## Discussion

### Efficacy of acupuncture

To our knowledge, this is the first systematic review and meta-analysis of English and Chinese literatures to investigate the efficacy of acupuncture for animal model on depressive disorder with behavioral changes as the outcome. The present study indicate that compared with western medicine, acupuncture has not less effect on behavior indicators including NC, NR, RSI, FW and GW for depression model rats, but the evidence on whether acupuncture is as effective as western medicine for depression model rats on CSI is insufficient. It demonstrates that acupuncture may have a potential disadvantage on improving the appetite of depression model rats in comparison with western medicine. Nevertheless, some researches showed that stimulations to acupoints of the stomach meridian such as Sibai (ST 2), Liangmen (ST 21), and Zusanli (ST 36) were effective in treating functional dyspepsia^98^, in which poor appetite is one of the core symptoms. In our analysis, Baihui (GV20) and Yintang (GV29) were most frequently used, while acupoints of stomach are scarcely used. Therefore, we consider that acupoints may be the main influencing factor in effecs of acupuncture on symptoms of depression.

### Explanations for indicators, models and interventions

In depression research, the OFT is a commonly indicator to assess the general locomotor activity and willingness to explore^9^. The SIT is a behavioral indicator that can assess the degree of anhedonia in models^10^. In addition, obvious change in weight is one of the core depressive-like symptoms. Moreover, some studies had doubt about the effects of acupuncture on these indicators in depression models^3, 7, 8^, which suggesting whether acupuncture has effects on these indicators needs to be resolved. Therefore, we selected NC, NR, CSI, RSI, FW and GW as behavioral indicators to evaluate the effects of acupuncture on depressive-like symptoms.

Successful animal models of depression should meet the following conditions^99^: (1) methods of modeling should have relations to pathogenesis of depression; (2) behavioral symptoms of models should be similar to clinical symptoms of depression; (3) the changes of pathophysiology in models should be similar to the changes of pathophysiology in depression; (4) antidepressant drugs are effective on behavioral symptoms of models; (5) behavioral symptoms of models should exist enough time for purpose of observing the effect of the treatment. A majority of studies in our analysis selected chronic stress models, which are most frequently used in depression experimental studies and considered to be with high validity, reliability and utility^100^, including CUMS, CUMS combined with solitary, CFSS^11^, CRS^26^, LH^39^, and PSA models^95^. Four studies made models on the basis of underlying pathogenesis of depression, included IA models^22^ (IA can simulate effects of the endogenous excitatory amino acids on the onset of depressive symptoms), L-AAA models (L-AAA can destroy astrocytes)^64^, EAAT1 antagonist models and EAAT2 antagonist models^77^ (EAAT1 and EAAT2 antagonist can inhibit functions of glutamate transporter). Results of these studies showed that the models were all successful. Nevertheless, it needs further researches to demonstrate which models have the highest validity, reliability and utility.

A Cochrane review has suggested that there is not enough evidence to demonstrate acupuncture is effective for depression and it has recommended future research should employ comparative designs and compare acupuncture with structured psychotherapies such as standard care^101^. Therefore, we compared acupuncture with western medicine, which can be considered as standard treatment in animal models at present. Current available anti-depressants are limited by lagged therapeutic time and undesirable side effects including acute nausea and headaches as well as chronic sexual dysfunction, weight gain, and diminished REM (rapid eyes movement) sleep^2^. We expected that acupuncture may have not less effect than anti-depressants on depression and have an advantage on less side effects^5, 102^, which is helpful for patients to adhere to the treatment.

### Mechanisms of acupuncture

The mechanisms of acupuncture on depression remain unclear. Main possible mechanisms of acupuncture include: (1) regulating the level of neurotransmitter such as monoamine neurotransmitter (5-HT, DA, NE^11, 71, 84, 93^), Glu and GABA; (2) regulating neuroendocrine system (HPA axise^88, 93^, HPT axise^52^, MT^59^); (3) regulating inflammatory cytokines such as IL-1β, IL-2, IL-6, TNF-α, NO, PGE2, iNOS, COX-2, NF-kB^21, 27, 49^; (4) promoting neuronal regeneration and neurotrophy through several signaling pathways (including BDNF, CREB, PKA, JNK, ERK, etc)^15, 19, 25, 32, 37, 46, 89^. In addition, acupuncture can modulate brain-gut peptide hormone (including GAS, NPY, CGRP, NT, etc)^55, 69^, inhibit over-activation of RAS and suppress oxidative stress^59, 62^. We consider that acupuncture treat depression by multi-targets through neuroendocrine-immune system and the future researches should enlarge the sample size and further clarify the pathophysiological effects of different acuopints or acupoint combinations.

### Inspirations for further studies

As shown in Table S1, acupoints that were most frequently used (over 10 studies) include scalp acupoints (GV20, GV29) and body acupoints (LR3, PC6, LI4, SP6). We expected that acupoints may be a potential source of heterogeneity among these studies. Hence, subgroup analysis according to different stimulation acupoints (scalp acupoints, body acupoints, or scalp acupoints and body acupoints) based on NC and GW was conducted (Table 4). Nevertheless, results showed that different stimulation acupoints may not be the source of heterogeneity. Due to some unexpected influences of acupuncture parameters (such as the stimulation dose of acupuncture, different meridians, and so on) and small sample size of included studies, it is difficult to deny acupoints’ contribution to the heterogeneity among the included studies.

It is also interesting to find that methods for selection of acupoints included on the basis of traditional Chinese medicine theory (49 studies), clinical experience (6 studies), preliminary clinical and/or experimental researches (19studies), summary of previous research reports (15 studies). This means that it doesn’t have a standard theoretical guidance for selection of acupoints. We suggest that future studies should clarify the physiological effects of different acupoints or acupoint combinations and the differences in their effects on depression.

Methodological quality of the included studies was generally low and only one of them got 6 points and two got 7 in our assessment. Randomization, blinding and sample-size estimation serve as the core standards of rigorous study design. In our analysis, only two studies did not describe randomization, but none of the studies described the sample size calculation, allocation concealment, blinded assessment of outcome. Blinding should also be utilized in intervention process. Research shows that the visual impact of needling is a potential factor that leads to the placebo effect of acupuncture^103^. Therefore, we suggest that animals should be prevented from seeing themselves or their companions being needled in intervention process. And more attention should be paid to the methodological quality in future studies.

In the sensitivity analysis, we considered that Jing2016^36^, Fan2013^21^ were the major sources of heterogeneity in NR, Zhang2008^88^, Fan2013^21^,Yang2013^81^ were the major sources of heterogeneity in CSI and Sun2016^66^ were the major sources of heterogeneity in RSI. We found that quality of Zhang2008^88^, Fan2013^21^ and Yang2013^81^ are considerable poor (one got 2 points and the other two got 3 points). We believe that avoiding publication bias and improving methodological quality of studies play an important role in reducing the heterogeneity in studies.

In the subgroup analysis, we did not find the exact factor that accounts for the heterogeneity among studies pooled in the meta-analysis of NC or GW. We also developed the meta regression and the result showed that factors of acupuncture treatment did not account for the heterogeneity in studies (Figs S11 and S12). More possible factors that can influence outcomes, such as experimental models, should be considered in later researches to interpret the heterogeneity among studies.

### Methodological interpretations

There were some methodological limitations in our study. First of all, we failed to meta-analyze all data because of insufficient data in several primary studies included. It is unclear whether the result would be changed or not when insufficient data were added in the analysis. Secondly, our study did not include data in other languages except for Chinese and English, which may result in certain degree of selective bias. Third, publication bias existed demonstrated by the asymmetry of the funnel plot and statistical analysis with Egger’s test. Some non-positive studies have been missed inevitably, as negative findings are less likely to be published. All the studies were published by Chinese authors, and only 1/9 of them were published in English journals. We cannot rule out the possibility that cultural difference is an underlying cause of publication bias. The above-mentioned methodological limitations suggested that the results should be interpreted with caution.

## Conclusions

Acupuncture has not less effect than western medicine on behavior indicators including NC, NR, FW, GW, and RSI. These indicators can be utilized to evaluate acupuncture effectiveness in experimental depressive disorder. Nevertheless, the results of this meta-analysis need to be interpreted with caution and high-quality researches are urgently needed. Future studies should pay more attention to the methodological quality, especially in sample size calculation, allocation concealment, blinding in intervention and assessment. Additionly, we suggest that future studies should further clarify the physiological effects of different acupoints or acupoint combinations and the differences in their effects on depression.

## Methods

### Search strategy

We searched the following databases from their inception up to January 13, 2017: PubMed, EMBASE, Cochrane Central Register of controlled trials(CENTRAL), China National Knowledge Infrastructure (CNKI), Wanfang data Information Site, and VIP information database. The search terms included ‘’, ‘’, ‘’, ‘’, ‘’, ‘’, ‘’ in Chinese and ‘electroacupuncture’, ‘acupuncture’, ‘depression’, ‘depressive’ in English. Besides, we also scanned the references of all the eligible studies carefully to identify further relevant publications.

### Eligibilit

Inclusion Criteria: (1) The objects are depressive models rat in both experimental and control group; (2) Interventions in experimental groups are acupuncture/electroacupuncture and in control groups are western medicine; (3) Outcome indicators should include at least one of the following indicators: number of crossings (NC) or number of rearings (NR) in open field test(OFT), capacity of sucrose intake (CSI) or rate of sucrose intake (RSI) in sucrose intake test (SI), final weight (FW) or gain weight (GW).

Exclusion criteria: (1) Experimental models combined depression with other diseases or disorders; (2) The western medicine control group is not set; (3) None of the indicators mentioned in inclusion criteria is reported; (4) Duplicate publications.

### Data extraction

We extracted the key contents of the studies in reference to the ARRIVE guideline^104^, and the following details were extracted: (1) publication year and the first author’s name; (2) animal species, sex, weight; (3) the randomization and blinding; (4) interventions and modeling; (5) outcome indicators. When studies set up two or more acupuncture groups, the group which had greater effects on the outcome indicators was extracted. All continuous data of outcome indicators which was presented as mean ± standard deviation (SD) were extracted if reported. Missing data or further information was sought from the primary authors via e-mail if necessary.

### Quality assessment

We evaluated the methodological quality of the included studies by applying the list of Collaborative Approach to Meta-Analysis and Review of Animal Data from Experimental Stroke (CAMARADES)^105^ which is modified on the basis of the characteristics of acupuncture treatment in depression researches when assessing the quality of studies, the items included: (1) sample size calculation; (2) randomization to treatment group; (3) allocation concealment; (4) blinded assessment of outcome; (5) correctness of methods of modeling; (6) avoidance of anesthetics with resistance to depressive; (7) describing control of temperature; (8) compliance with animal welfare regulations; (9) publication in a peer-reviewed journal; (10) declared any potential conflict of interest. For the calculation of an aggregate quality score, each item was attributed one point. Two authors independently extracted data and assessed study quality. Disagreements were solved after discussion over the details of the studies.

### Statistical Analysis

Meta-analysis and subgroup-analysis were performed by RevMan V.5.3, and analysis of publication bias and Meta-regression were conducted with STATA/SE 12.0. We considered all behavioral indicators as continuous data, and then an estimate of the combined effect sizes utilizing standard mean difference (SMD) with the given random effects model. We used the random model rather than fixed since heterogeneity between multi-studies has to be taken into account. Publication bias was assessed with a funnel plot and Egger’s test. To assess heterogeneity, the I^2^ statistic was used. To clarify the impact of factors potentially modifying the outcome measures, we also conducted sensitivity analysis and subgroup analysis according to the following variables: different types of acupuncture, different stimulation acupoints, different intervention time, different duration of treatment. The difference between groups was assessed by partitioning heterogeneity and using the χ^2^ distribution with n-1 degrees of freedom (df), where n indicate the number of groups. Probability value p < 0.05 was considered significant.

### Data Availability

All data generated or analysed during this study are included in this published article (and its Supplementary Information files).

## Electronic supplementary material

Supplement materials Table S1, Figure S1-S12
