# Supplementary material for: Acupuncture for behavioral changes of experimental depressive disorder: a systematic review and meta-analysis
Source: Sci Rep. 2017 Aug 29;7:9669. doi: 10.1038/s41598-017-09712-1 (PMC5575352; doi:10.1038/s41598-017-09712-1)
Supplement: Supplementary file 1 — Supplement materials Table S1, Figure S1-S12 [file 41598_2017_9712_MOESM1_ESM.doc]

**Acupuncture for behavioral changes of experimental depressive disorder: a systematic review and meta-analysis**

Ren-zhong Kou^1^*, Hong Chen^1^*, Mei-ling Yu^1^, Tian-cheng Xu^1^, Shu-ping Fu^1,2^, Sheng-feng Lu^1,2☆^

1. The No.2 Clinical Medicine College, Nanjing University of Chinese Medicine, Nanjing, 210023, China.
2. Key Laboratory of Acupuncture and Medicine Research of Ministry of Education, Nanjing University of Chinese Medicine, Nanjing, 210023, China.

* These authors contributed equally to this work.

☆Corresponding to A/Prof. Sheng-feng Lu at: 138 Xianlin Road, Qixia District, Nanjing, Jiangsu 210023, China. Tel: +86-25-85811234; fax: +86-25-85811234;

E-mail address: lushengfeng@njucm.edu.cn (SFL)

**Supplement materials**

**Table S1. Acupoints and methods for selection of acupoints of the studies included**

| Research | Acupoints | Methods for Selection of Acupoints |
| --- | --- | --- |
| Bao 2014^11^ | GV20,GV29 | not mention |
| Bao 2014#^12^ | GV20,GV29 | not mention |
| Chen 2011^13^ | GV20,GV24 | traditional Chinese medicine theory |
| Cheng 2015^14^ | GV20,GV29 | not mention |
| Dai 2010^15^ | GV20,GV29 | not mention |
| Deng 2013^16^ | GV20,GV29 | traditional Chinese medicine theory;  summary of previous research reports |
| Ding 2016^17^ | GV20,GV14 | traditional Chinese medicine theory |
| Duan 2008^18^ | GV20,GV29,EX-HNl | traditional Chinese medicine theory |
| Duan 2016^19^ | GV20,GV29 | not mention |
| Duan 2016#^20^ | GV20,GV29 | not mention |
| Fan 2013^21^ | EX-HNl,PC6,SP6 | clinical experience |
| Fan 2016^22^ | LI4,LR3 | not mention |
| Fan 2016#^23^ | LI4,LR3 | not mention |
| Fan 2016*^24^ | LI4,LR3 | not mention |
| Fu 2008^25^ | LI4,LR3 | not mention |
| Guo 2016^26^ | GV20,GV29,SP6 | preliminary clinical and experimental researches |
| Hu 2013^27^ | GV20,PC6 | traditional Chinese medicine theory;  summary of previous research reports |
| Hu 2014^28^ | GV20,GV29 | traditional Chinese medicine theory;  summary of previous research reports |
| Huang 2005^29^ | PC6,CV17 | traditional Chinese medicine theory;  preliminary experimental researches |
| Research | Acupoints | Methods for Selection of Acupoints |
| Ji 2013^30^ | GV20,GV29 | traditional Chinese medicine theory |
| Jia 2005^31^ | GV20,GV29 | traditional Chinese medicine theory;  summary of previous research reports |
| Jiang 2007^32^ | GV20,GV29 | traditional Chinese medicine theory;  summary of previous research reports;  preliminary experimental researches |
| Jiang 2013^33^ | LI4,LR3 | traditional Chinese medicine theory;  preliminary clinical researches |
| Jiao 2008^34^ | GV20,GV29 | traditional Chinese medicine theory;  preliminary experimental researches |
| Jin 2015^35^ | GV20,PC6 | traditional Chinese medicine theory |
| Jing 2016^36^ | GV20,GV29 | traditional Chinese medicine theory;  summary of previous research reports |
| Li 2007^37^ | GV20,GV29 | not mention |
| Li 2008^38^ | GV20,GV29 | not mention |
| Li 2011^39^ | GV20,GB34 | not mention |
| Li 2011#^39^ | GV20,GB34 | not mention |
| Li 2011&^40^ | GV20,GV29 | not mention |
| Li 2014^41^ | GV20,GV29 | not mention |
| Lin 2008^42^ | GV20,SP6 | traditional Chinese medicine theory;  summary of previous research reports |
| Liu 2005^43^ | GV20,GB34 | not mention |
| Liu 2008^44^ | GV20,GB34 | traditional Chinese medicine theory;  summary of previous research reports;  preliminary experimental researches |
| Liu 2009^45^ | GV20,GV29,ST36,SP6 | not mention |
| Liu 2012^46^ | LI4,LR3 | not mention |
| Lu 2008^47^ | GV20,GV29,ST36,ST40 | traditional Chinese medicine theory |
| Lu 2013^48^ | GV20,PC6 | not mention |
| Research | Acupoints | Methods for Selection of Acupoints |
| Lu 2016^49^ | GV20,PC6 | not mention |
| Lu 2016#^50^ | GV20,PC6 | not mention |
| Luo 2016^51^ | LI4,LR3 | not mention |
| Ma 2016^52^ | GV20,GV29 | not mention |
| Mo 2014^53^ | GV20,GV29 | traditional Chinese medicine theory;  preliminary experimental researches |
| Pan 2016^54^ | EX-HN1、PC6、SP6 | traditional Chinese medicine theory |
| Qin 2010^55^ | GV20,GV29,ST25 | not mention |
| Shao 2016^56^ | GV20,PC6 | traditional Chinese medicine theory;  summary of previous research reports;  preliminary experimental researches |
| Shi 2007^57^ | GV20,GV29 | traditional Chinese medicine theory;  preliminary experimental researches |
| Shi 2015^58^ | GV20,GV29,GB20,BL23 | traditional Chinese medicine theory;  preliminary clinical and experimental researches |
| Song 2014^59^ | GV20,GV29 | traditional Chinese medicine theory;  summary of previous research reports |
| Song 2015^60^ | GV20,GV29 | traditional Chinese medicine theory;  summary of previous research reports;  preliminary experimental researches |
| Song 2016^61^ | GV20,GV14 | traditional Chinese medicine theory |
| Song 2014#^62^ | GV20,GV29 | traditional Chinese medicine theory |
| Sun 2003^63^ | GV20,ST36 | traditional Chinese medicine theory |
| Sun 2013^64^ | LI4,LR3 | traditional Chinese medicine theory |
| Sun 2014^65^ | GV20,GV29,PC6 | not mention |
| Sun 2016^66^ | CV4，ST36 | summary of previous research reports |
| Tang 2013^67^ | GV20,GV29 | traditional Chinese medicine theory |
| Tang 2014^68^ | GV20,GV29 | not mention |
| Research | Acupoints | Methods for Selection of Acupoints |
| Teng 2013^69^ | GV20,GV29 | traditional Chinese medicine theory |
| Wang 2008^70^ | GV20,GV29,SP6 | traditional Chinese medicine theory |
| Wang 2009^71^ | GV20,GV29 | traditional Chinese medicine theory;  summary of previous research reports;  preliminary experimental researches |
| Wang 2010^72^ | GV20,LR3 | traditional Chinese medicine theory;  summary of previous research reports |
| Wang 2013^73^ | GV20,GV24 | not mention |
| Wang 2014^74^ | Ex-B2 | traditional Chinese medicine theory |
| Wang 2016^75^ | GV20,GV24 | traditional Chinese medicine theory;  preliminary experimental researches |
| Wu 2007^76^ | ST36 | preliminary experimental researches |
| Xiao 2014^77^ | LI4,LR3 | traditional Chinese medicine theory;  clinical experience |
| Xiao 2014#^77^ | LI4,LR3 | traditional Chinese medicine theory;  clinical experience |
| Xiao 2014&^78^ | LI4,LR3 | traditional Chinese medicine theory |
| Xiao 2014*^77^ | LI4,LR3 | traditional Chinese medicine theory;  clinical experience |
| Xu 2016^79^ | GV20,GV29 | not mention |
| Xu 2016#^80^ | GV20,GV29 | traditional Chinese medicine theory;  preliminary clinical and experimental researches |
| Yang 2013^81^ | ST36 | traditional Chinese medicine theory |
| Yang 2013#^82^ | GV20,GV29 | not mention |
| Yu 2006^83^ | GV20,GB34 | not mention |
| Yu 2012^84^ | GV20,GV29 | traditional Chinese medicine theory;  preliminary clinical and experimental researches |
| Yu 2016^85^ | GV20,GV29 | traditional Chinese medicine theory;  preliminary clinical and experimental researches |
| Yu 2006#^86^ | GV20,Anmian | not mention |
| Research | Acupoints | Methods for Selection of Acupoints |
| Zhang 2005^87^ | GV20,PC6,GV24,SP6 | not mention |
| Zhang 2008^88^ | GV20,GV29 | not mention |
| Zhang 2016^89^ | GV20,GV29 | not mention |
| Zhang# 2016^90^ | GV20,PC6、SP6 | traditional Chinese medicine theory;clinical experience |
| Zhang 2016&^91^ | LI4,LR3 | traditional Chinese medicine theory;clinical experience |
| Zhang 2016*^92^ | GV20,GV29 | traditional Chinese medicine theory;  summary of previous research reports;  preliminary clinical and experimental researches |
| Zhao 2005^93^ | GV20,SP6 | traditional Chinese medicine theory |
| Zheng 2013^94^ | GV20,GV24,EX-HNl | not mention |
| Zhou 2008^95^ | GV20,PC6,GV24,SP6 | traditional Chinese medicine theory |
| Zhu 2015^96^ | GV20,Anmian | preliminary experimental researches |
| Zhuang 2007^97^ | GV20,BL15,BL18 | traditional Chinese medicine theory |

**Notes:** **Contents in brackets include names of acupoints and the frequency of the selections.** GV20 (Baihui, 71 studies), GV29 (Yintang, 44 studies), LR3(Taichong, 14 studies), PC6 (Neiguan, 14 studies), LI4 (Hegu, 13 studies), SP6 (Sanyinjiao, 11 studies), GV24 (Shenting, 6 studies), ST36 (Zusanli, 6 studies), EX-HNl (Sishencong, 5 studies), GB34 (Yanglingquan, 5 studies), ST25 (Tianshu, 2 studies), GV14 Dazhui, 2 studies), Anmian (2 studies), CV17 (Danzhong, 1 studies), CV4 (Guanyuan, 1 studies), BL15 (Xinshu, 1 studies), BL18 (Ganshu, 1 studies), GB20 (Fengchi, 1 studies), ST40 (Fenglong, 1 studies), EX-B2 (Jiaji, 1 studies).


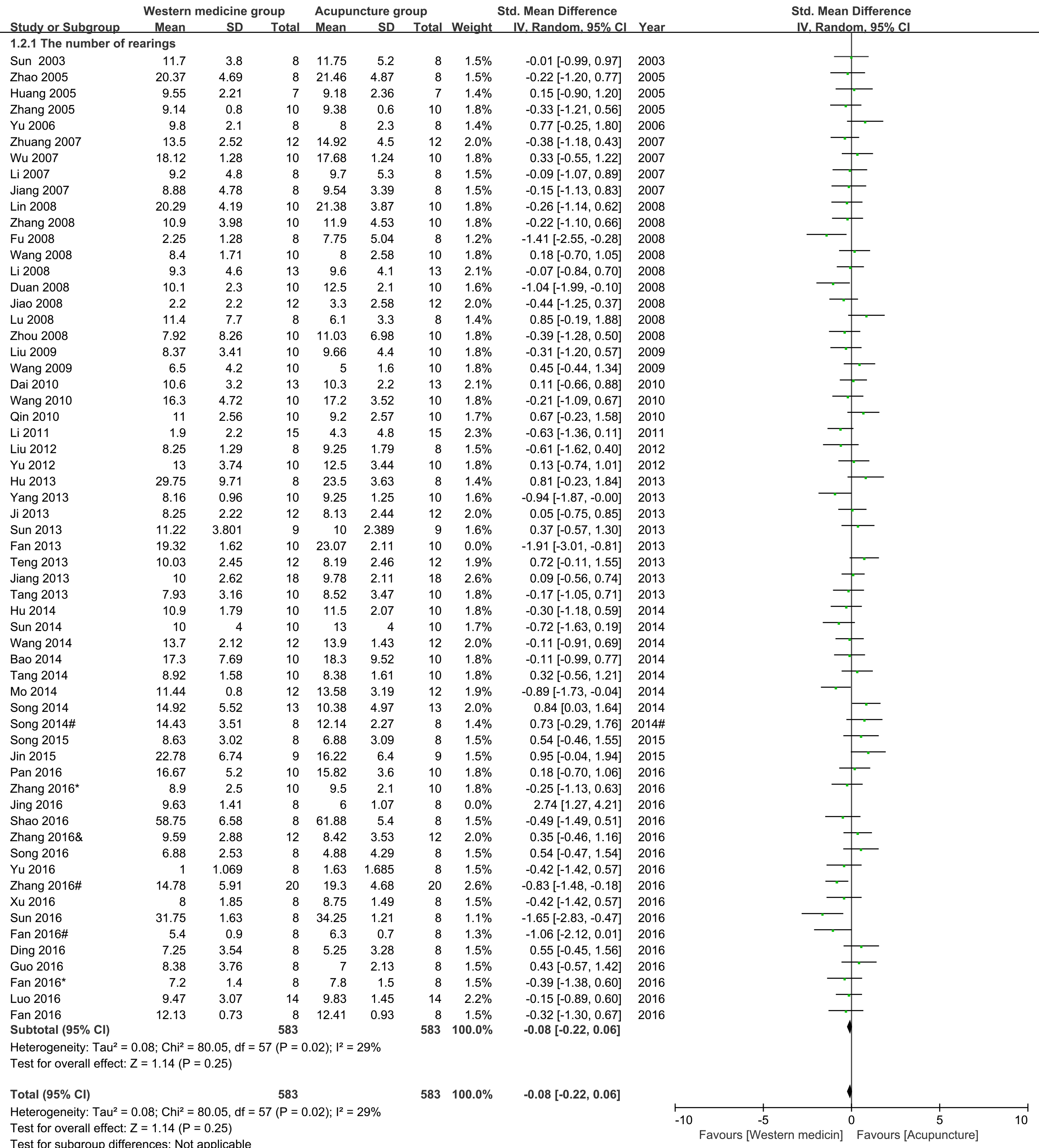


**Figure S1. heterogeneity of NR after removing Jing2016, Fan2013 in the analysis**


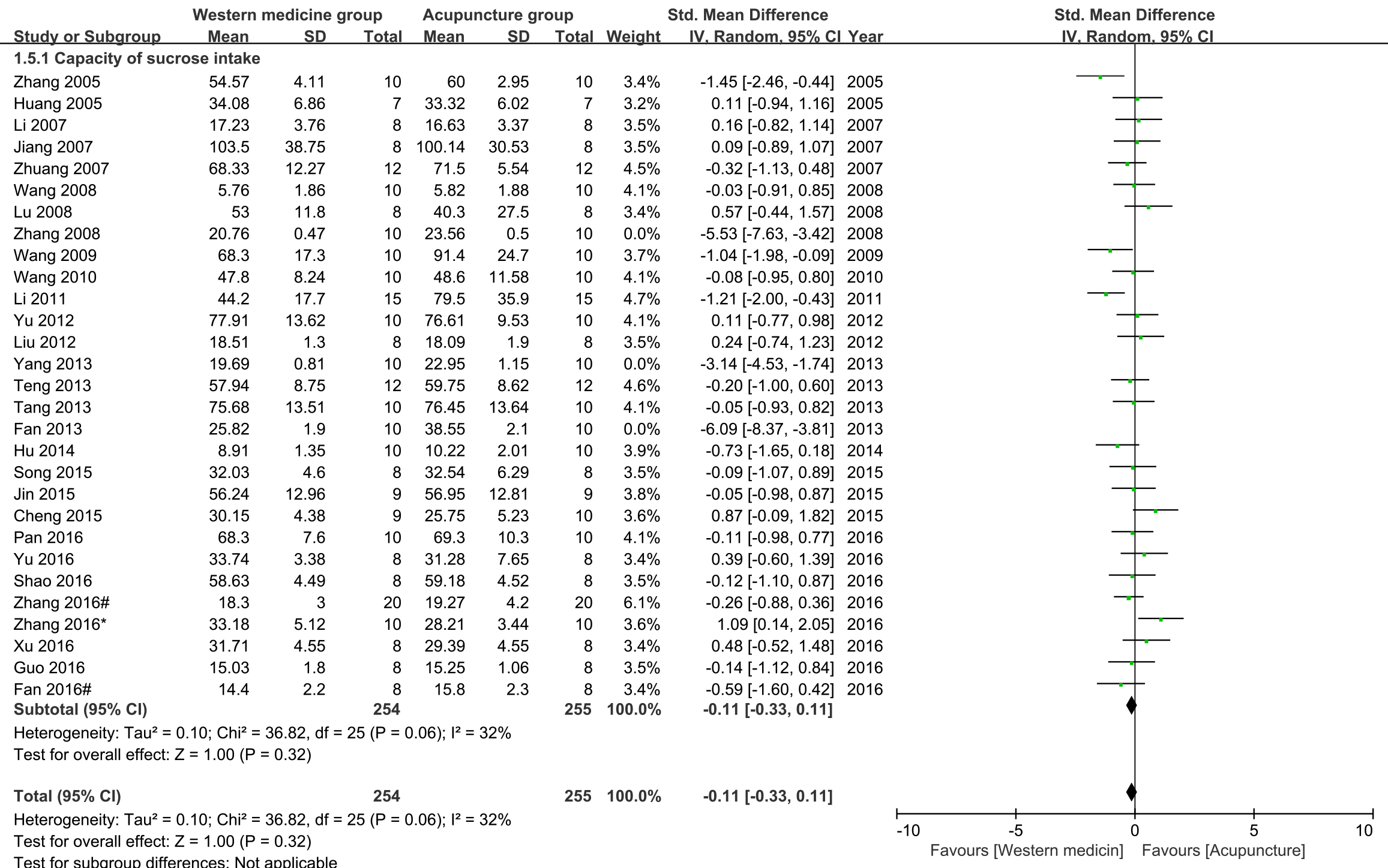


**Figure S2. heterogeneity of CSI after removing Zhang2008, Fan2013,Yang2013 in the analysis.**

**
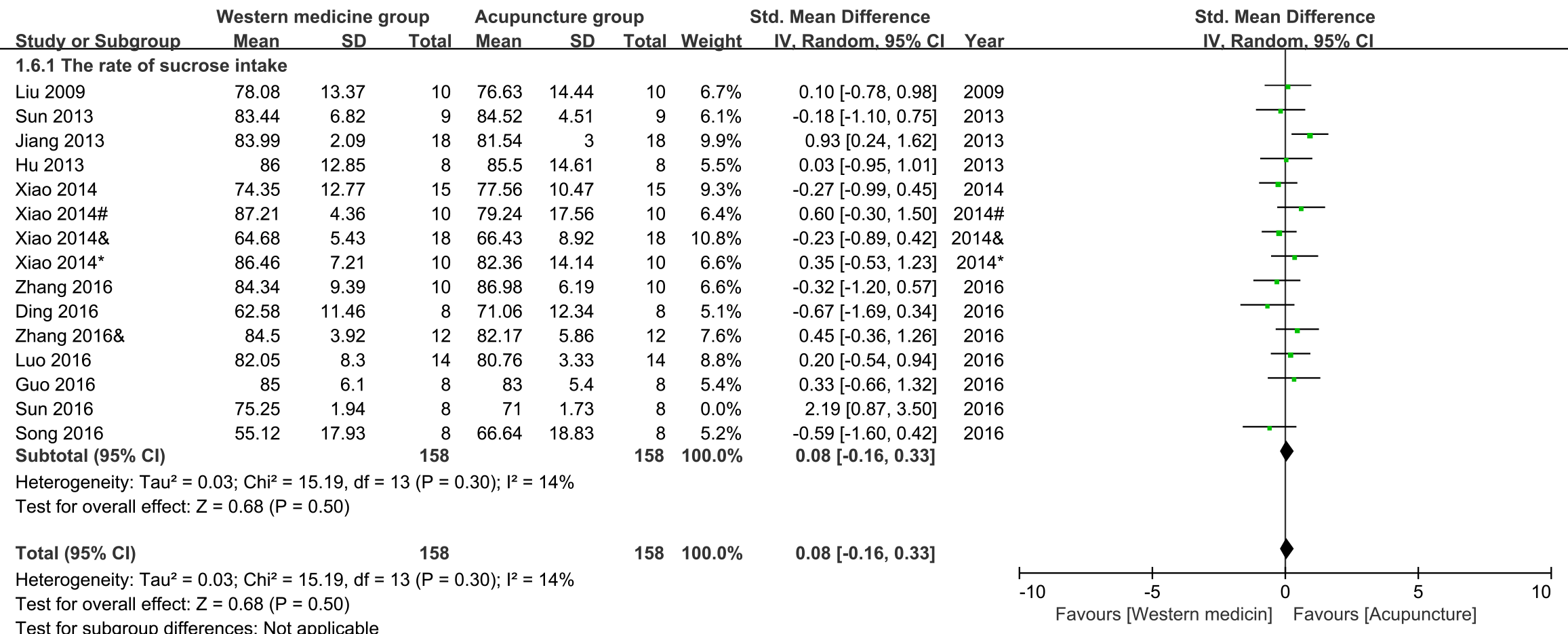
**

**Figure S3. heterogeneity of RSI after removing Sun2016 in the analysis.**

**
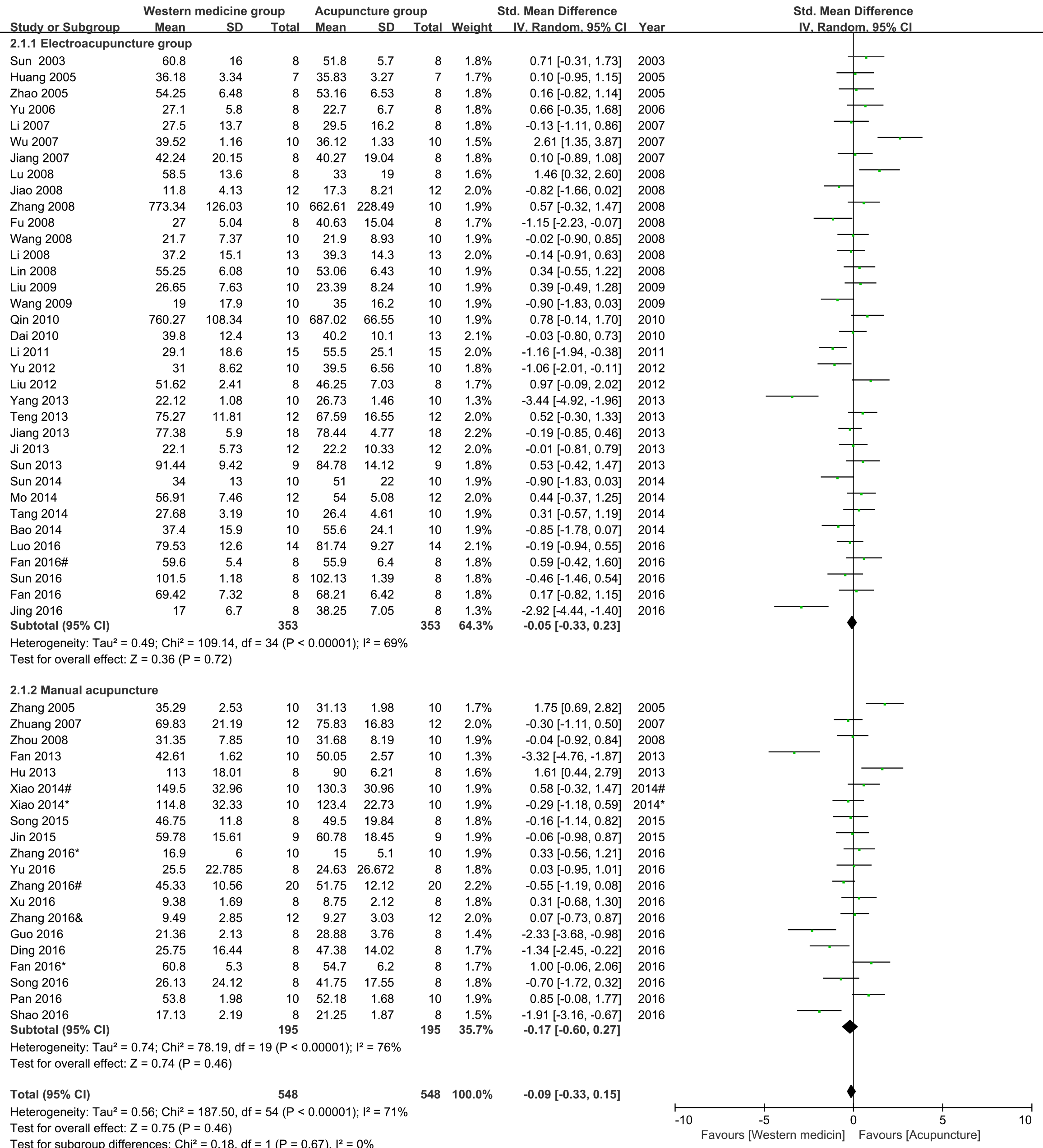
**

**Figure S4.Subgroup analysis of NC according to different types of acupuncture.**

**
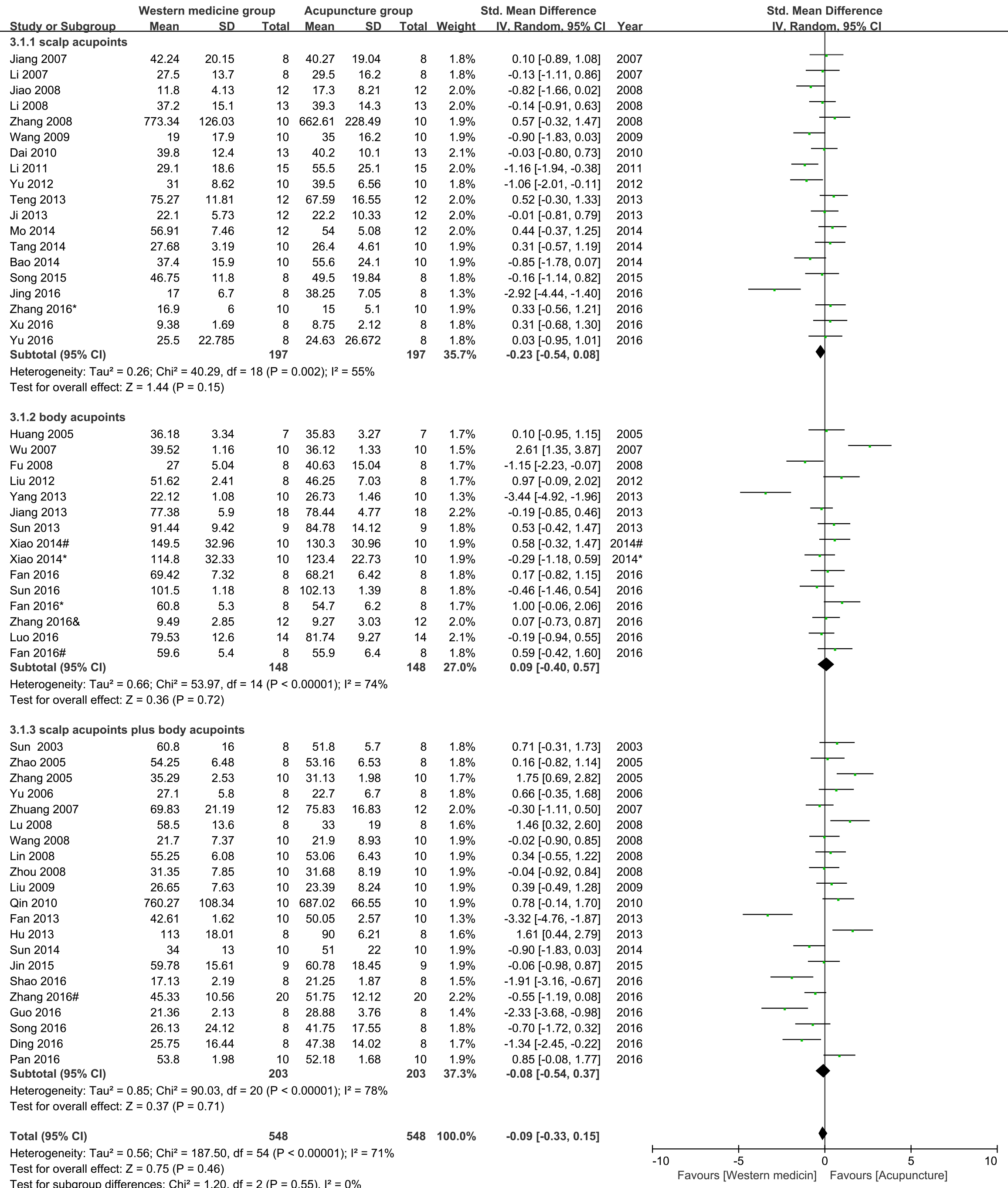
**

**Figure S5.Subgroup analysis of NC according to different stimulation acupoints.**

**
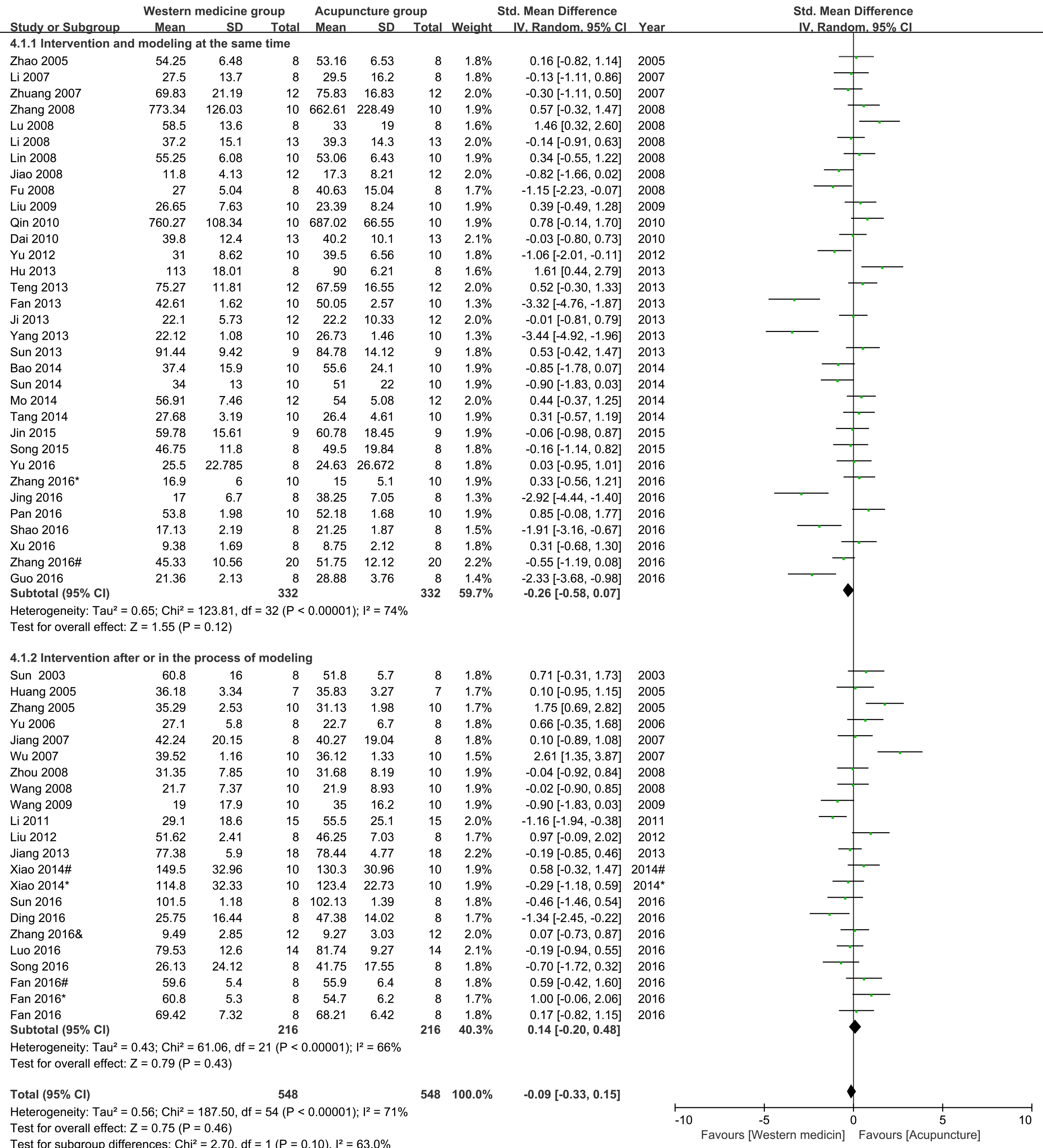
**

**Figure S6.Subgroup analysis of NC according to different intervention time.**

**
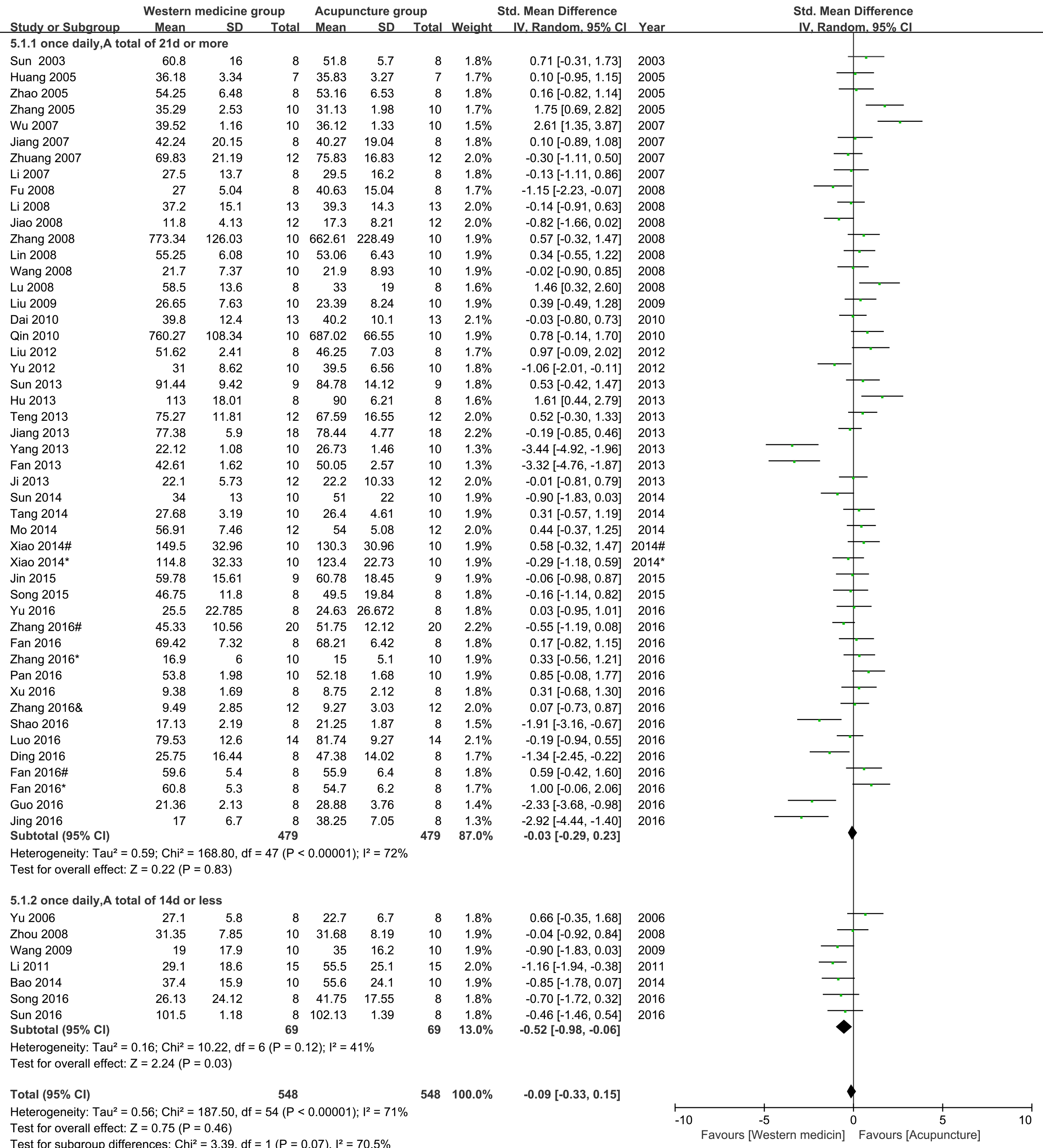
**

**Figure S7.Subgroup analysis of NC according to different intervention duration.**

**
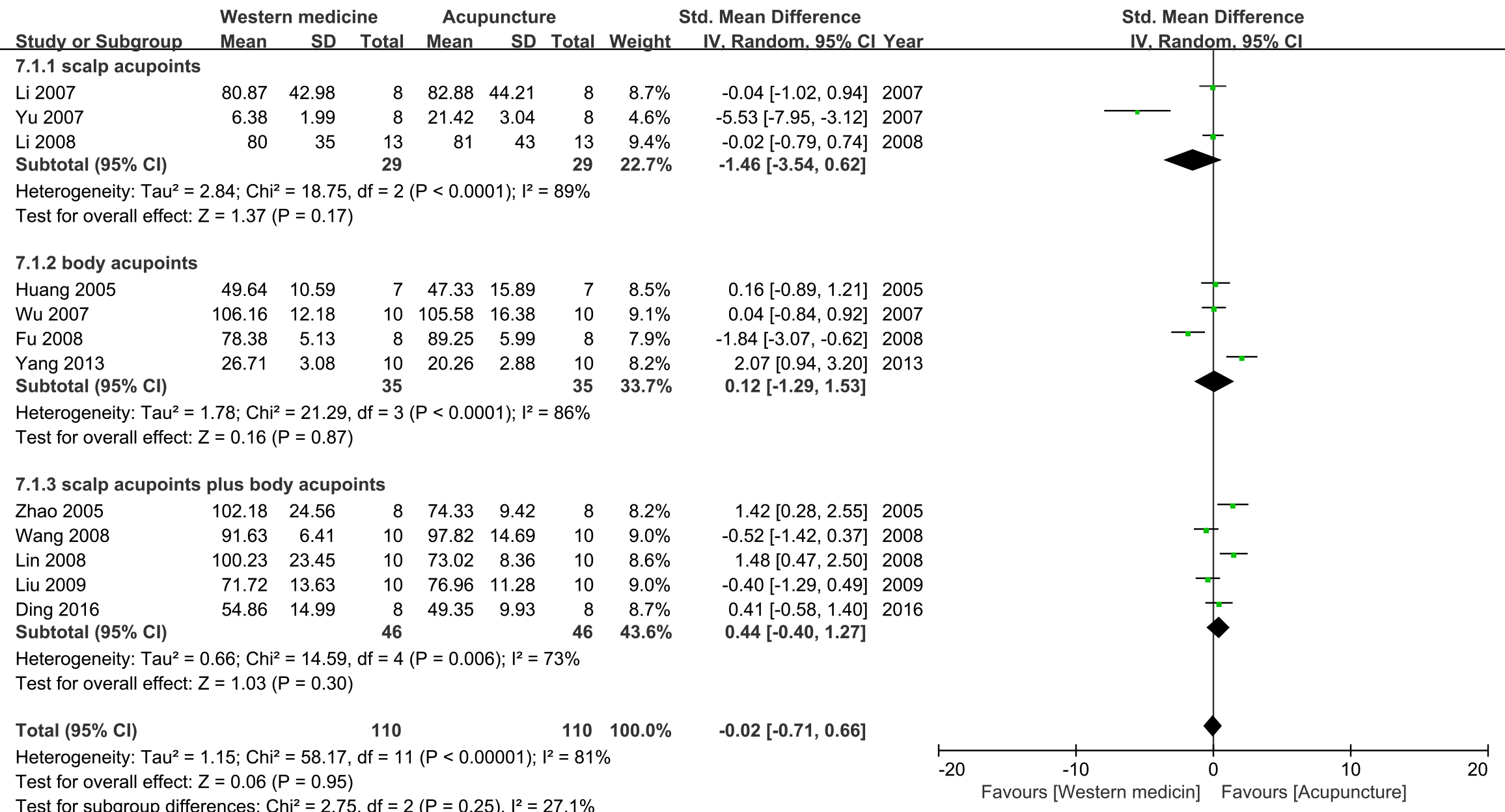
**

**Figure S8.Subgroup analysis of GW according to different stimulation acupoints.**

**
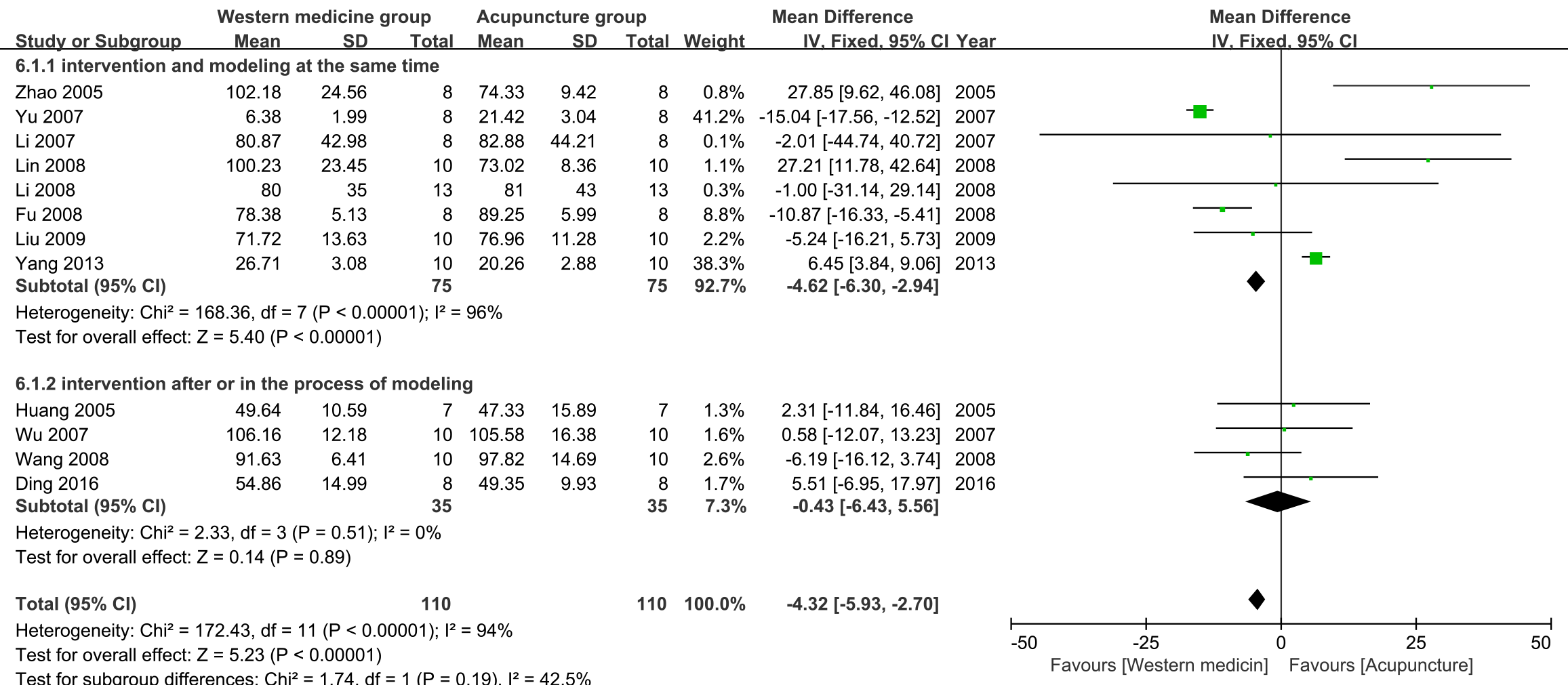
**

**Figure S9.Subgroup analysis of GW according to different intervention time.**


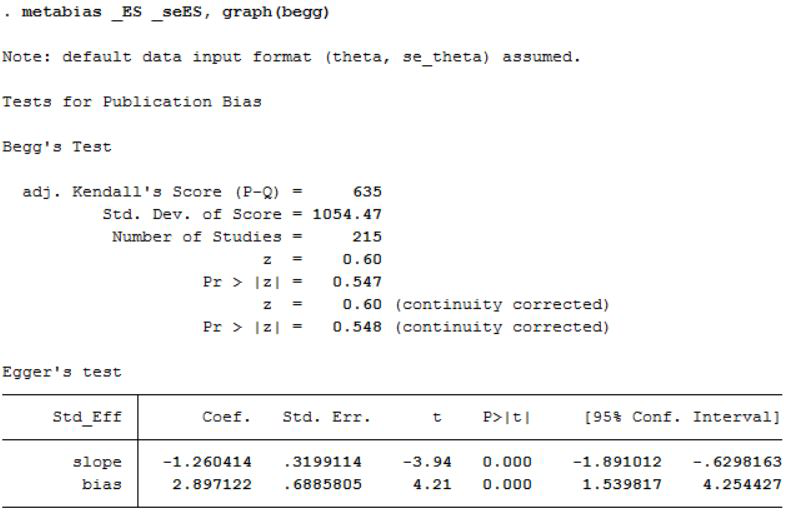


**Figure S10. Publication bias analyzation**

**
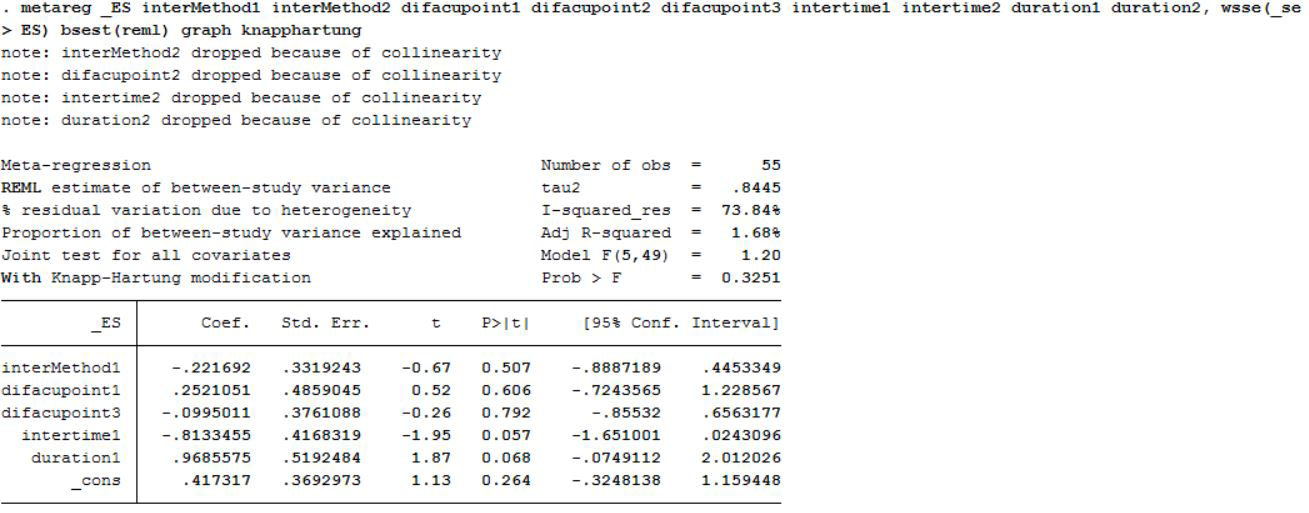
**

**Figure S11. Meta-regression of NC according to factors influencing the effect of acupuncture**


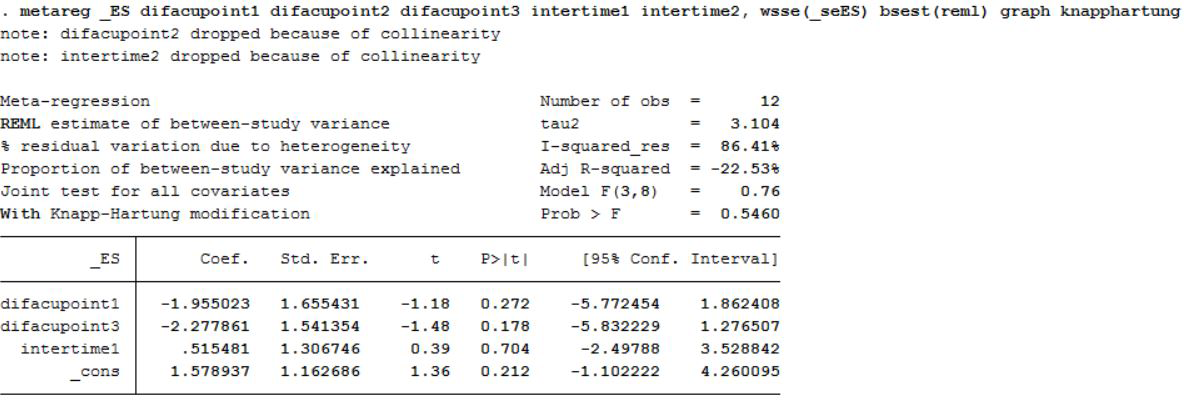


**Figure S12. Meta-regression of GW according to factors influencing the effect of acupuncture**
